# Correlations of mobility and Covid-19 transmission in global data

**DOI:** 10.1371/journal.pone.0279484

**Published:** 2023-07-19

**Authors:** Nittai K. Bergman, Ram Fishman

**Affiliations:** 1 Berglas School of Economics, Tel Aviv University, Tel Aviv, Israel; 2 Department of Public Policy, Tel Aviv University, Tel Aviv, Israel; National Taiwan University, TAIWAN

## Abstract

Assessing the contribution of mobility declines to the control of Covid-19 diffusion is an urgent challenge of global import. We analyze the temporal correlation between transmission rates and societal mobility levels using daily mobility data from Google and Apple in an international panel of 99 countries during the period of March-December 2020. Reduced form regression estimates that flexibly control for time trends suggest that globally, a 10 percentage point reduction in mobility is associated with a 0.05–0.07 reduction in the value of the effective reproduction number, *R*(*t*). However, the strength of the association varies substantially across world regions and over time, being initially positive and strong in most world regions during the 2020 spring period, but becoming weaker over the summer, especially in Europe and Asia. We further find evidence that the strength of the association between mobility and transmission rates is reduced where facial coverings rules were implemented.

## Introduction

By some estimates, more than a third of the global population have been subjected to severe mobility restrictions since the start of the Covid-19 pandemic. Numerous governments around the world have resorted to such “lockdowns” as their primary strategy of limiting the transmission of infection, at enormous economic and social costs. As costs escalate, and transmission rates decline in some countries, a growing debate has emerged regarding when and how lockdowns should be eased, and whether it is possible to do so without unleashing additional waves of infection. An assessment of the relation between mobility levels and transmission rates can be of value in helping to navigate this policy dilemma and in understanding the determinants of diffusion.

Multiple papers have estimated the declines in transmission rates that occurred following lockdowns and other non pharmaceutical interventions (NPI) by using detailed case-level data in specific localities or multiple countries [1–10]. In this paper, we use publicly available data to empirically estimate the relation between transmission rates (effective reproduction numbers) and societal mobility levels using a large, international 126-country panel, covering the period between late February and December 2020 (later periods are excluded from the analysis because the appearance of new variants can bias the results). Our assessment employs a reduced-form regression analysis based on daily mobility data provided by Google and Apple, and estimates of daily transmission rates at the country level from Arroyo-Marioli et al. [10].

While studying the effect of lockdowns is clearly important, we focus on the association between transmission rates and mobility rather than on the association between transmission rates and government issued Nonpharmaceutical Interventions (NPIs), and in particular, lockdown orders. We do so for three reasons. The first is that evidence suggests an imperfect correspondence between lockdowns and mobility levels, with mobility declining prior to lockdowns (or even in their absence), and in certain cases increasing prior to formal lockdown easing [11]. Second, as governments consider the degree to which lockdown conditions can be eased, it is important to analyze the span of the relation between mobility and transmission rates and not only the effects of discrete, large reductions in mobility resulting from lockdown orders. As one example, government ordered lockdowns may have acted as a signaling device indicating the severity of the health crisis, thereby influencing individual behavior. Finally, lockdowns may impact transmission rates not solely due to their direct effect on mobility rates, but also through their impact on other forms of individual behavior. Future changes in mobility levels need not necessarily come about in tandem with such changes in individual level behavior.

Several other papers have also analyzed the relation between mobility and Covid-19 transmission rates at a wide range of scales [12–17]. This study analyzes the relation at a global sample covering 126 countries, and complements the approaches of previous studies by employing established, reduced-form, panel data econometric methods. These methods do not depend on process-based modeling choices, and enable progress on two gaps in the literature highlighted in a recent review [18]. First, they enable us to separate the impacts of variation in mobility from other confounding variables that vary across countries, or regionally over time, and may also affect transmission rates. Second, they easily lend themselves to estimating not only the magnitude of the relation between mobility and transmission rates, but also how that relation changes in locations and times in which other NPIs (such as facial covering, for example) are introduced by governments. From a policy point of view, understanding how government interventions affect the relation between mobility and transmission rates is of paramount importance, since they may allow countries to resume economic activity without increasing transmission rates substantially.

**Fig 1:**
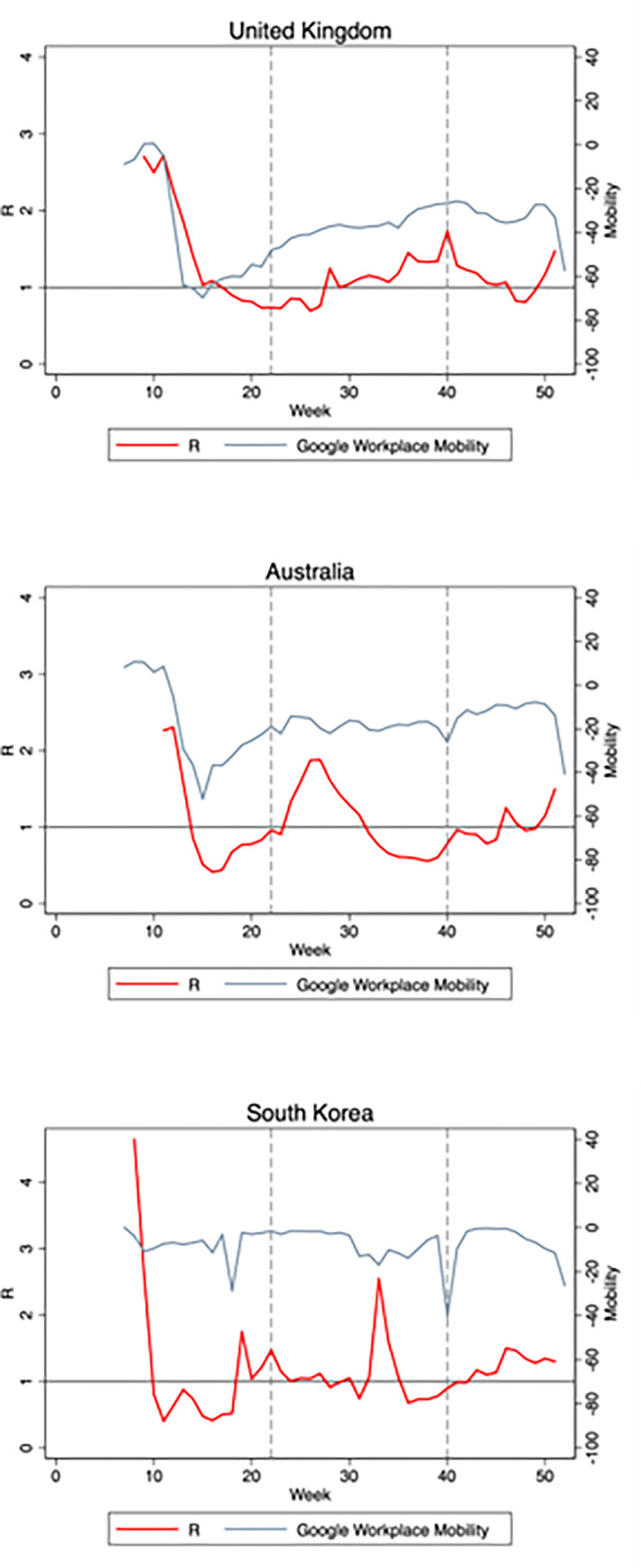
Plots of estimated effective reproduction numbers *R*(*t*) and Google workplace mobility indicators for the U.K., Australia and South Korea. *R*(*t*) is plotted against the left axis. Mobility changes from baseline (in percentage points, see data section) are plotted against the right axis. The vertical lines represent the partition of the study period into the three sub-periods discussed in the main text.

A visual inspection of the country level data suggests mixed patterns regarding the relation between transmission rates and mobility. In the U.K, for example (Fig 1, top panel), transmission rates seem to track mobility levels quire consistently throughout the year. In Australia (middle panel), transmission rates also seem to track mobility levels initially. Steep reductions in mobility are followed by significant reductions in transmission rates, while subsequent to a rise in mobility commencing mid-April, transmission rates rise as well. However, later on, transmission rates decline without a corresponding decline in mobility. In South Korea (bottom panel), there is no clear indication of a consistent relation between transmission rates and mobility levels.

Our reduced form regression analysis is designed to estimate the average correlation between mobility and transmission rates in a large international sample of countries. By providing such summary estimates at global scale, our analysis complements important studies that analyze sub-national transmission dynamics and underlying epidemiological processes at finer resolution.

## Data and methods

### Mobility data

The principal measure of mobility used in this analysis is taken from the *Covid 19 Community Mobility Reports* provided by Google. We also use the *Mobility Trends Reports* provided by Apple as a robustness test (see SOM). The Google data utilize anonymized location-based information to assess changes in the number of visits to several categories of locations in a given date and country, as compared to a baseline value for that day of week. The baseline period is the median value for the corresponding day of week, calculated during the 5-week period Jan 3–Feb 6, 2020. The categories include retail and recreation, groceries and pharmacy, parks, transit stations, workplaces, and residential. (see https://www.google.com/Covid19/mobility/) It is available for 132 countries. We averaged mobility values at the weekly level in each country in order to smooth high frequency variation. S1 Fig in S1 File plots Google data on visits to workplaces over time, averaged in six world regions. S2 Fig in S1 File plots variation in mobility over time and by country, grouped into regions, and shows there is a substantial degree of variation in mobility dynamics across countries, even though some broad patterns are shared across countries in the same region. S3 Fig in S1 File plots the six measures of Google mobility data over time, averaged over Europe.

### Covid-19 transmission rates

The preferred indicator of Covid-19 transmission rates is the effective reproduction number *R*(*t*), which measures the number of individuals an average infected person infects during the period of infection. In the primary analysis, we make use of country level estimates of *R*(*t*), provided by reference [10] between January 23rd and Dec 31st for 177 countries (temporal coverage varies by country and begins after 100 cases are confirmed). To construct this proxy, data on new cases, recoveries, and deaths is used to back out estimates of *R*(*t*) on the basis of disease models [10]. Importantly, the data (and the estimates) are smoothed with Kalman-filtering techniques. This means that discrete, high frequency movements in the actual effective reproduction number will be difficult to observe in these estimates. In addition, the estimates do not account for the delay between actual infection and official diagnosis. As such, they reflect lagged infection rates, with a lag size that combines the delay between infection and Covid-19 testing and the time between testing and official reporting of test results. Previous studies report an average incubation period of 5 days [19,20]. In our analysis we assume an overall lag of 2 weeks, although our results are not sensitive to using a lag of 1 week.

As is well known, a major limitation shared by all proxies based on confirmed case counts is that they are likely to substantially underestimate the true number of cases in the population. To the extent that the ratio of confirmed to actual cases is constant within countries (even if not between countries), however, this will not bias the *R* estimates. Further, the estimation method is argued to be robust even when new cases are imperfectly measured, or the true dynamics of the disease do not follow the SIR model [10].

S4 Fig in S1 File plots R(t) estimates over time, averaged in six world regions. After declining from very high initial levels during the early phase of the pandemic, in the spring, R(t) varies across regions, and shows a prominent second wave in Europe and North America over the fall.

### Sample

S5 Fig in S1 File reports the number of mobility and R observations (i.e. countries for which data is available) by week. The main sample in our analysis includes 4,782 observations (week-country combinations) from 126 countries (displayed in S6 Fig in S1 File) over the period March 13th—Dec 31st when using Google data. Data coverage is uneven across countries, starting when the confirmed number of cases reaches 100 in each country.

### Empirical strategy

We employ standard panel-regression techniques to estimate the association between transmission rates (proxied by the *R* estimates described above) and mobility measures. A similar reduced-form methodology has been used to study the correlation between the growth rate of Covid-19 infections and non-pharmaceutical interventions [7]. The regressions include country specific fixed effects (intercepts) to flexibly account for all time-invariant country attributes, thus basing estimates of the relation between transmission rates and mobility on the correlation between these two variables over time within countries.

Formally, we estimate the following baseline regression:

$$T_{ct}=\mu M_{c,t-2}+\alpha_{c}+\beta_{t}+\varepsilon_{ct}$$ (1)

where *T* is a proxy for Covid-19 transmission rates in country *c* (in region *r*) on week *t* as described above, and *M* is one of the mobility measures described above, measured 1 week before the date at which *T* is observed. The regression include country fixed effects *α_c_* and week fixed effects *β_t_*. In a robustness test, we replace the global week fixed effects with world-region specific week fixed effects *β_rt_*. In another test, we include regional fixed effects for the time (in weeks) which have elapsed since the 100th case in each country. Because transmission and mobility may exhibit temporal autocorrelation within countries, all standard errors are clustered at the country level.

Since mobility measures are strongly correlated temporally within countries (S3 Fig in S1 File), separating out the effects of each type of mobility indicator demands statistical power that is unlikely to be provided by the current sample. Our main regression models therefore include a single measure of societal mobility as the explanatory variable. We focus on the Google workplace mobility measure, being a natural proxy for economic activity.

We also estimate a related first-differences model in which both the outcome (transmission) and explanatory (mobility) variables are replaced with their changes compared to the previous week:

$${(T}_{ct}-T_{c,t-1})=\mu (M_{c,t-2}-M_{c,t-3})+\alpha_{c}+\varepsilon_{ct}$$ (2)

This more demanding first-difference model helps examine the robustness of a time series model in general, and estimates whether short-term changes in mobility translate to short-term changes in infection rates in the same manner that variation over flexible time scales does (as captured in Eq 1).

### General trends in mobility and transmission

**Fig 2:**
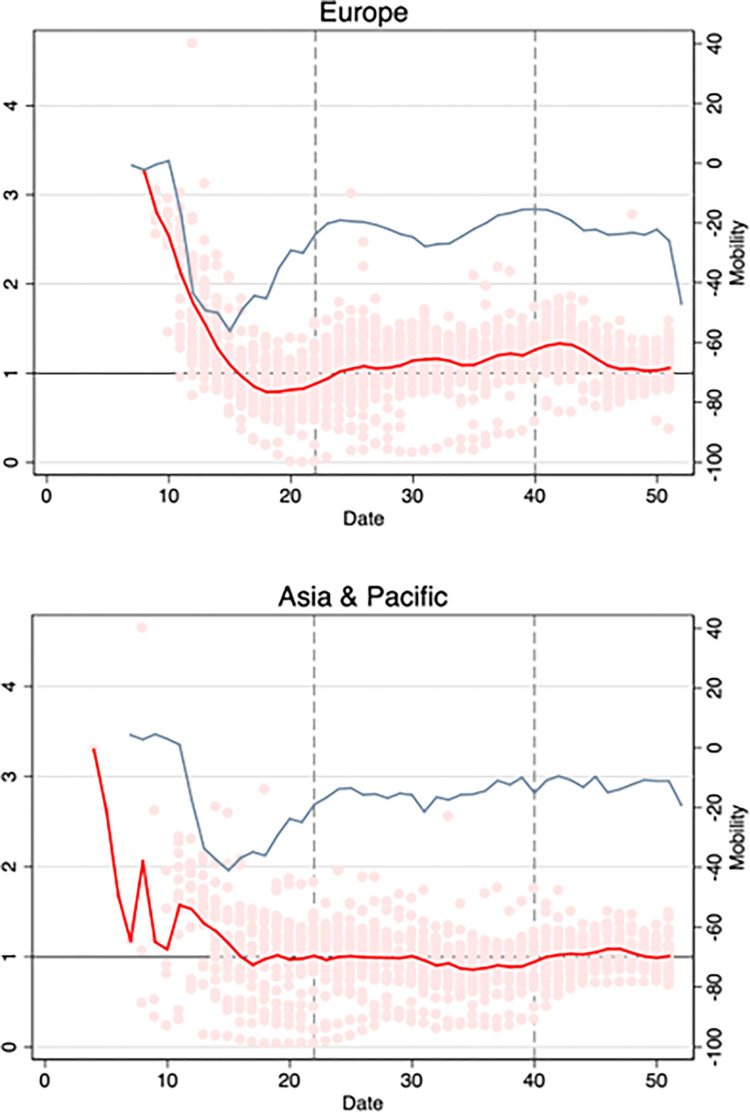
Plots of *R*(*t*) (red line) and Google (visit to workplaces) mobility (blue line) indicators over time, averaged for Europe and Asia. *R*(*t*) is plotted against the left axis. Mobility changes from baseline (in percentage points, see data section) are plotted against the right axis. Red circles represent country specific values of R(t). The vertical lines represent the partition of the study period into the three sub-periods discussed in the main text.

Fig 2 (top panel) exhibits the European averages of Google workplace mobility and (unlagged) *R*(*t*) estimates over time. During the earlier part of the sample period, the data exhibit significant downward trends in both mobility and estimated transmission rates. As can be seen, European mobility levels decline sharply between mid-February and March 2020 (with Google workplace mobility declining by approximately 50 percentage points) and bottom in April. Over the same time period, Fig 2 (top panel) shows a decline in estimated *R* values from 3.5 to below approximately 0.8. Mobility then rises until June, when it begins declining again, albeit not as strongly. During that time, R(t) rises as well, until fall. Starting in August, mobility rises again until about November and then begins declining again.

Fig 2 (bottom panel) provides analogous information for countries in the sample within the “Asia and Pacific” region. Similar to Europe, the figure indicates a decline of average *R* of approximately 3.1 units from a peak of 4 to a value of 0.9, and a decline of 40 percentage points in the Google workplace mobility measure. However, from May onwards, mobility recovers, although not nearly fully, whereas R(t) remains quite stable and close to the threshold value of R = 1.

Changes in average mobility levels mask a good deal of country-level heterogeneity (S2 Fig in S1 File). Examining regional patterns only serves as motivation for the full sample country-level analysis which follows below.

**Fig 3:**
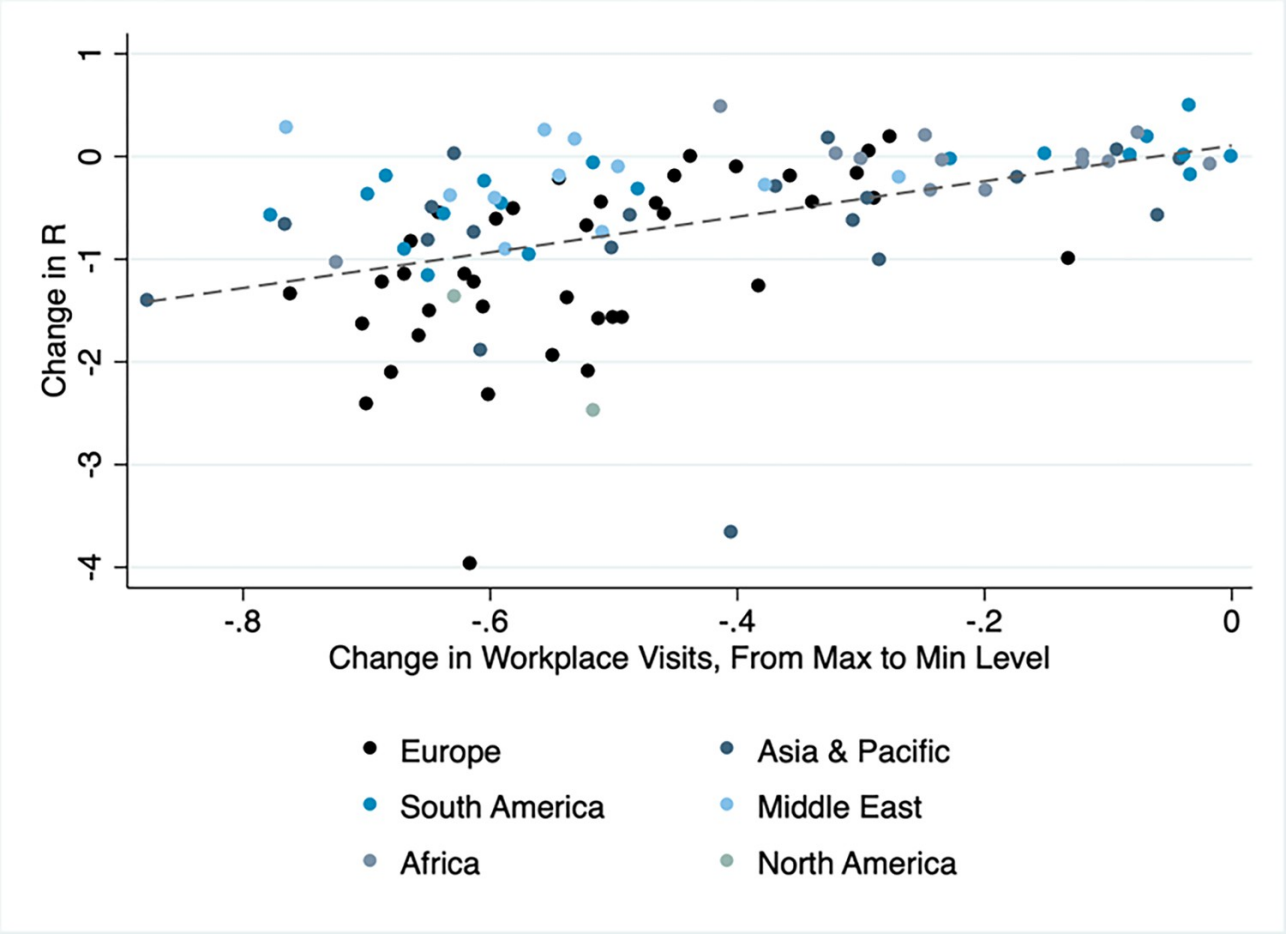
Scatter plot of country level changes in R values vs. the change in Google workplace mobility from its maximum (initial) to its minimum level. Marker colors indicate world region. The dashed line represents estimates of a linear regression.

In Fig 3 we plot, on the horizontal axis, the decline in Google workplace visits for each country in our sample, from its highest level, mostly occurring around mid March, to the lowest value reached in each country, mostly occurring in April. In many of these countries, visits to workplace mobility decline by 50–70 percentage points (p.p.), with Spain leading in Europe with an almost 80 p.p. decline. On the vertical axis, we plot the change in *R*(*t*) over the same time period in each country, which ranges from 0–2.5 units, depending on the country.

There is a clear positive relation between the two trends: countries which exhibit larger decreases in Google workplace visits, also, on average, show a greater decline in *R*(*t*). This is signified by the fitted trend line, which has a statistically significant positive slope of b = 0.17 units of *R* per 10 p.p. in workplace visits (p<0.001). The relation is stronger in Europe (b = 0.31, p<0.001) than in Asia (b = 0.1, p<0.05) and South America (b = 0.1, p<0.001), and statistically insignificant in Africa and the Middle East.

Overall, the data show that countries which reduced workplace visits to a greater extent, on average, also reduced transmission rates more (as discussed above, this does not necessarily signify a causal relation). In the following section, we investigate the correlation between changes in *R*(*t*) and workplace mobility more formally and over the entire period extending until the end of 2020.

## Regression analysis results

**Fig 4:**
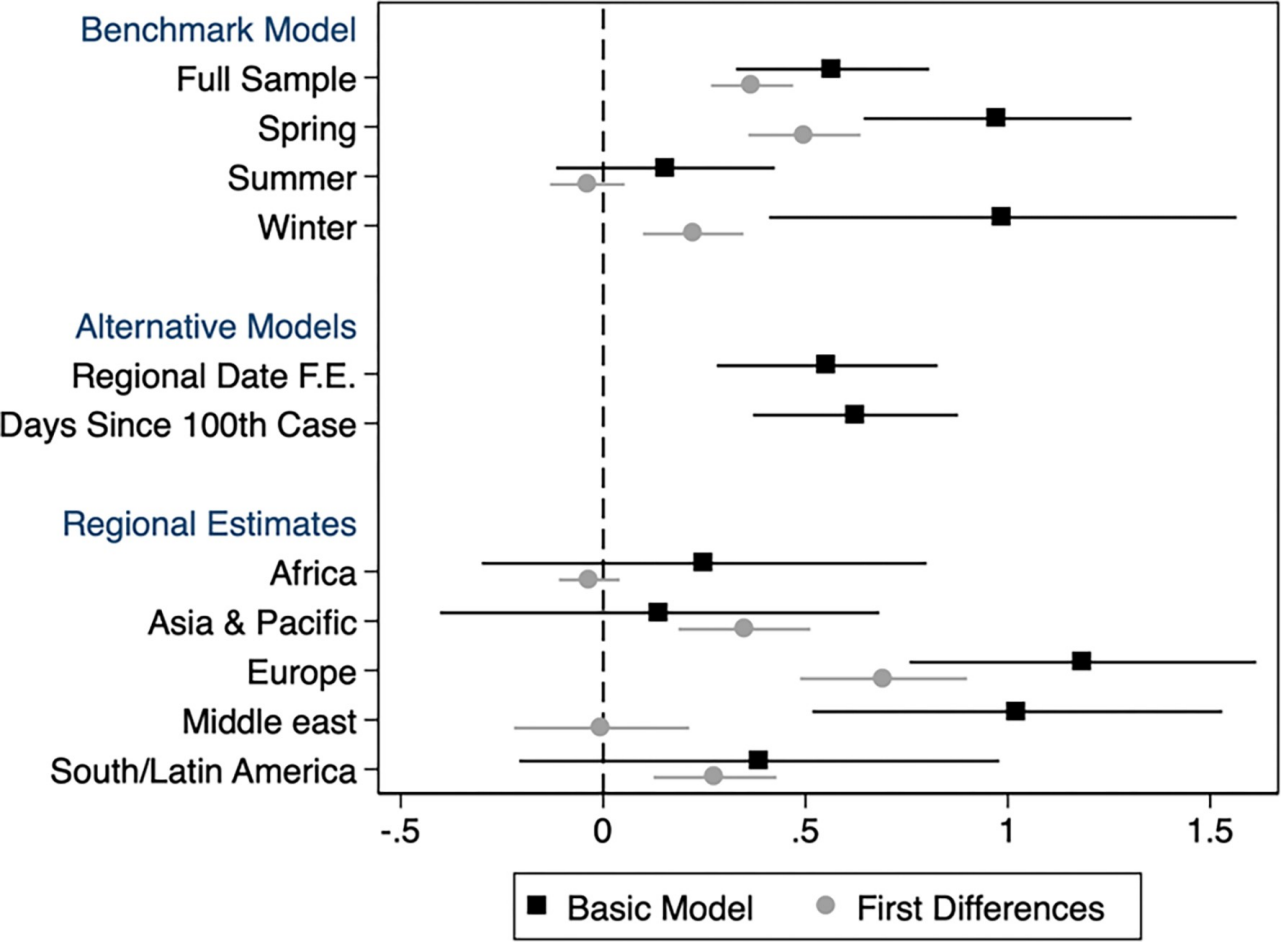
Coefficients from regressions of estimated effective reproduction numbers *R*(*t*) observed at the country-date level on Google workplace mobility indicators (see text for details). Black markers capture estimates from variants of the basic regression model, whereas Grey markets indicate estimates from parallel first-differences regressions. The benchmark model includes country and date fixed effects. Alternative models include additional controls as specified. Error bars indicate 95% confidence intervals corresponding to standard errors that are clustered at the country level.

Estimates of regression (1) are plotted in Fig 4, and tabulated in Tables 1–3.

**Table 1:** Baseline regression results relating estimated *R* values to two weeks lagged Google workplace mobility.

	(1)	(2)	(3)	(4)	(5)
	R	R	R	R	R
Google Workplace Mobility (Seven-day Lagged)	0.68***	0.57***	0.55***	0.62***	0.37***
	(0.10)	(0.12)	(0.14)	(0.13)	(0.05)
Observations	4782	4782	4744	4743	4651
Adjusted R-squared	0.311	0.463	0.506	0.499	0.035
Week F.E.	None	Global	Regional	Global	N
Weeks Since 100th Case F.E.	N	N	N	Regional	N

Each column reports results from a separate regression. Regressions in columns 1–4 include country fixed effects. Column 5 reports estimates of a first-difference model. Standard errors, clustered by country, are reported in parentheses. Stars indicate statistical significance

(* p<0.1

** p<0.05

*** p<0.01).

**Table 2:** Regional regression results relating estimated *R* values to 2 weeks lagged Google workplace mobility.

	(1)	(2)	(3)	(4)	(5)
	R	R	R	R	R
	**Africa**	**Asia &** **Pacific**	**Europe**	**Middle East**	**South/ Latin** **America**
	Google Workplace Mobility				
Mobility (7-day Lagged)	0.25	0.14	1.19***	1.02***	0.39
	(0.27)	(0.26)	(0.21)	(0.23)	(0.29)
Observations	807	883	1598	508	866
Adjusted R-squared	0.558	0.379	0.554	0.309	0.512

Each column report results from a separate regression. All regressions include country fixed effects. Standard errors, clustered by country, are reported in parentheses. Stars indicate statistical significance

(* p<0.1

** p<0.05

*** p<0.01).

**Table 3:** Regression results relating estimated *R* values to 2 weeks lagged Google workplace mobility in three periods: Spring (March to May), Summer (June to September) and Winter (October to December).

	(1)	(2)	(3)
	R	R	R
	**Spring**	**Summer**	**Winter**
	Google Workplace Mobility		
Mobility (7-day Lagged)	0.97***	0.15	0.99***
	(0.17)	(0.14)	(0.29)
Observations	1206	2188	1386
Adjusted R-squared	0.657	0.521	0.616

Each column report results from a separate regression. All regressions include country fixed effects and week fixed effects. Standard errors, clustered by country, are reported in parentheses. Stars indicate statistical significance

(* p<0.1

** p<0.05

*** p<0.01).

Table 1 provides results of regressions that use estimates of the effective reproduction number (*R*) from [10] as the outcome variable, and a 2-week lagged measure of workplace mobility from Google data. Google mobility data is coded here as the fraction decline from baseline levels. For example, a 10 percentage point reduction in mobility is coded as -0.1. The coefficients should therefore be interpreted as the associated decline in the effective reproduction number associated with a 100 percentage point (p.p) decline in mobility. All specifications include country fixed effects (intercepts) to account for all cross-country differences in transmission stemming from time invariant country characteristics. To account for time trends, the specification in Column 2 (Table 1) includes global date fixed effects and the specification in Column 3 includes region-by-date fixed effects. The specification in Column 4 includes country-specific fixed effects for each possible value of the number of weeks elapsed since it confirmed its 100th Covid-19 case. Column 5 reports estimates of the first-differences model (Eq 2).

Across the four specifications (Columns 1–4) reported in Table 1, we estimate a positive and statistically significant relation between effective reproduction numbers, *R*, and Google mobility levels, indicating that increased mobility is associated with increased transmission. The coefficient on lagged mobility ranges from 0.55 to 0.68, depending on the specification. The estimates imply that a ten percentage point drop in the Google mobility measure is associated with a decline of between 0.05–0.07 units of *R*, depending on the specification used. The first-differences model, which is based on short-term weekly variation alone, yields a somewhat smaller estimate of 0.37.

As additional robustness tests, S1 Table in S1 File reports similar estimations to Table 1 which, however, use alternative measures of transmission other than R(t), such as the growth in the number of cases. S2 Table in S1 File reports parallel estimates to those reported in Table 1 but which make use of Apple mobility data instead of Google mobility data.

S7 Fig in S1 File (top panel) plots the week specific intercepts (fixed effects) estimated in regression (1), which reflect the average global weekly variation in transmission that is unexplained by mobility reductions or by time-invariant country specific factors. As can be seen, the weekly fixed effects decline over time during the spring, indicating increased global suppression of transmission rates stemming from measures unrelated to variation in mobility (these measures could potentially include increased usage of masks, increased hygiene, favorable weather trends, etc); but then exhibit two additional “waves” over the course of the year, albeit of much smaller magnitude than the first one. S7 Fig in S1 File (middle panel) plots the country-level fixed effects estimated through regression (1). The figure provides a measure of the variation in the reduction in transmission rates across countries that is unexplained by mobility levels. The figure also plots (bottom panel) the fixed effects of a variant of specification (1) which uses region, as opposed to country, fixed effects (estimated in relation to the European value). A clear ranking emerges in regions’ ability to reduce transmission rates using non-mobility suppression methods, with Asia most successful, North America least successful, and European countries in between (the omitted regional fixed effect is Europe).

Table 2 reports estimates derived separately in five different geographical regions. As there are only two countries in the North America region in our data, we do not run the regression separately for that region. As can be seen, the positive relation between transmission rates and mobility levels is concentrated in Europe and the Middle East regions, where a 10 p.p. reduction in mobility is associated with more than a 0.1 decline in the value of R. No statistically significant relation between transmission rates and lagged mobility is observed in the other regions. To estimate R, it is required that the number of reported Covid-19 cases exceed 100 [10], implying that countries where infection rates climbed in later time periods are underrepresented in the sample.

Table 3 reports estimates derived separately in three different sub-periods: the “Spring” (March-May), “Summer” (June-September) and “Winter” (October-December), as indicated by the vertical lines in Figs 1 and 2. The separation is motivated by seasonal effects and by the occurrence of the main waves of the pandemic in Europe and North America. The estimates indicate strong associations in the spring and winter periods, which are remarkably similar in magnitude, and a much smaller and insignificant association over the summer.

**Fig 5:**
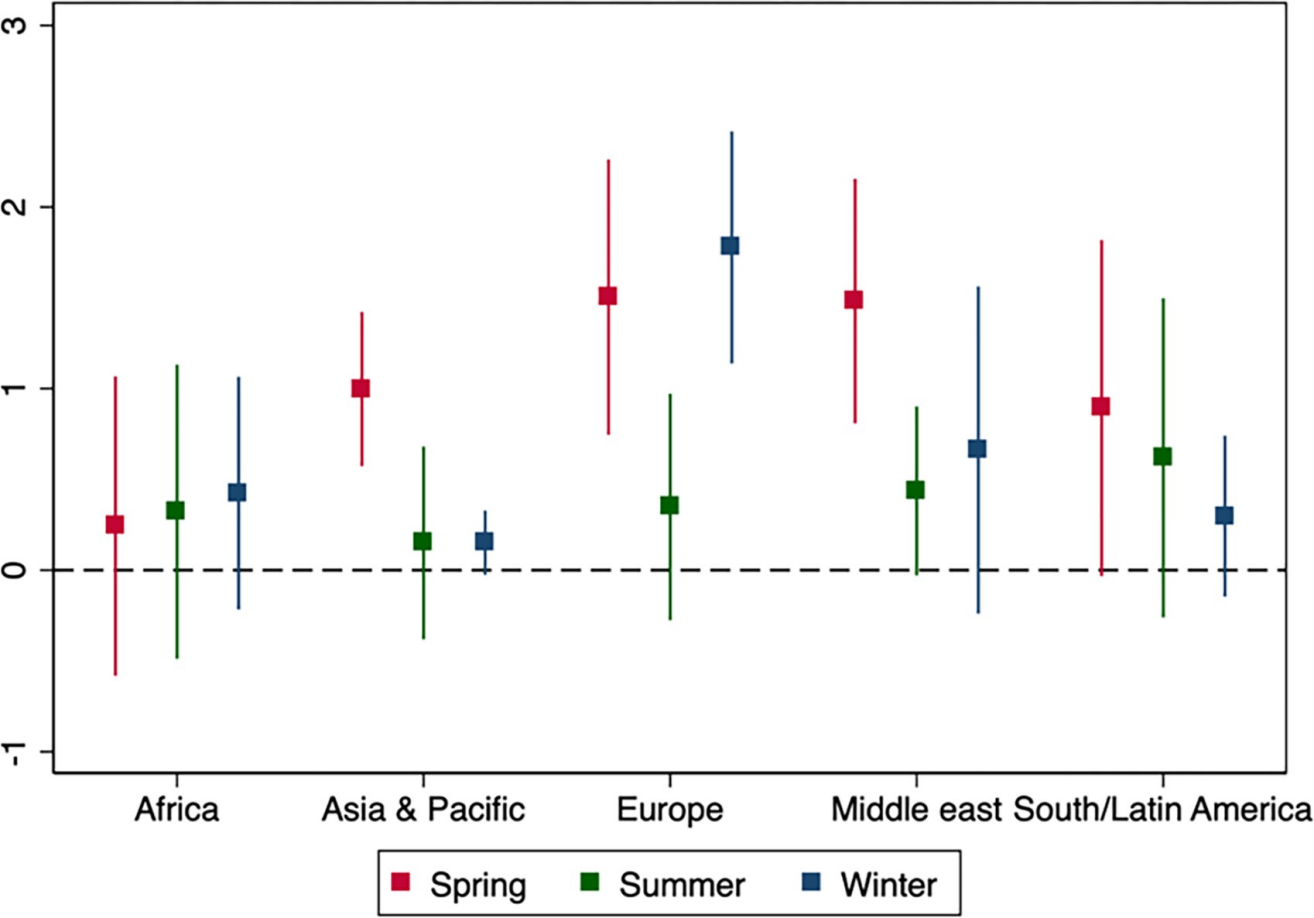
Coefficients from regressions of estimated effective reproduction numbers R(t) observed at the country-week level on Google workplace mobility indicators (see text for details). Markers indicate estimates from variants of the basic regression model at specific regions and periods of the year (Spring: March to May, Summer: June to September, Winter: October-December). All regressions include country and week fixed effects. Error bars indicate 95% confidence intervals corresponding to standard errors that are clustered at the country level.

The results are summarized in Fig 4, which displays estimates for the main and the first-differences models (black and gray markets, respectively) side by side in the different periods (top panel) and regions (bottom panel) of the sample. Fig 5 offers greater detail, and plots regional estimates in each of the three sub-periods. During the spring period, we find positive associations in all five regions, and all but in Africa are statistically significant. Other than in Africa, where estimates remain stable over time, we see a reduction in the association between transmission and mobility into the summer, especially in Asia, Europe and the Middle East. However, with the onset of winter, the association remains low in Asia but bounces back in Europe to even exceed its original strength.

## Government actions and the mobility-transmission relation

We have seen above that there is a great deal of variation in the strength of the mobility-transmission relation across countries and over time. A natural question to ask is whether other forms of behavior change or limitation can help reduce the strength of the relation. From a policy point of view, measures that would help reduce the strength of the relation are of paramount importance, since they may allow countries to resume economic activity–at least insofar as mobility to workplaces is concerned–without increasing transmission rates substantially.

To examine this question empirically, we make use of detailed data on a range of responses undertaken by governments to the Covid-19 outbreak at a daily time scale [21] by the Oxford COVID-19 Government Response Tracker (OxCGRT). The data attributes a severity index to a range of potential responses by country and date. Responses are grouped into four types, of which the type of greatest interest for our purpose are health system policies, which consist of public information campaigns, contact tracing and facial coverings. Data on containment and closure policies are also of potential interest, as they are designed to reduce mobility. In results which are omitted for brevity, we find that these policies indeed reduce mobility [21]. These are the measures in the data set which may help induce protective behavior by individuals, thus potentially reducing the magnitude of the mobility-transmission relation. When incorporating this data into the analysis, our sample is slightly reduced to include 4642 observations from 121 countries.

To estimate the degree to which these results are effective, we estimate an interaction model of the form:

$$T_{ct}=\mu M_{c,t-2}+{\nu R}_{c,t-2}+\lambda M_{c,t-2}\times R_{c,t-2}+\alpha_{c}+\beta_{t}+\varepsilon_{ct}$$ (3)

which is similar to regression (1) except that it includes a term for a government intervention R and its interaction with mobility, all estimated with a two week lag. OxCGRT data encodes R with a severity index detailed in Table 4, with 0 indicating no such action was taken, and higher integer numbers between 1–4 indicating progressively severe action. Even though data is available for the three types of responses mentioned above, in practice, we only use data on contact tracing and facial coverings, since public information campaigns are imposed by the great majority of governments throughout the study period, leaving little variation to use in the estimation. While the interaction regression yields strong and large effects, we prefer to avoid reporting a result which is based on a very small sample size of countries which did not implement public information campaigns.

**Table 4:** Codes for government action variables used in the analysis, adapted from the OxCGRT website (https://github.com/OxCGRT/covid-policy-tracker/blob/master/documentation/codebook.md).

Response	Description	Values
Public Information Campaign	Record presence of public info campaigns	0—no Covid-19 public information campaign 1—public officials urging caution about Covid-19 2- coordinated public information campaign (eg across traditional and social media)
Contact Tracing	Record government policy on contact tracing after a positive diagnosis Note: we are looking for policies that would identify all people potentially exposed to Covid-19; voluntary bluetooth apps are unlikely to achieve this	0—no contact tracing 1—limited contact tracing; not done for all cases 2—comprehensive contact tracing; done for all identified cases
Facial Covering	Record policies on the use of facial coverings outside the home	0—No policy 1—Recommended 2—Required in some specified shared/public spaces outside the home with other people present, or some situations when social distancing not possible 3—Required in all shared/public spaces outside the home with other people present or all situations when social distancing not possible 4—Required outside the home at all times regardless of location or presence of other people

Table 5 reports estimates of regression (3) for the two relevant types of government interventions, i.e. facial coverings (panel A) and contact tracing (panel B). In these estimates, R is taken as a categorical variable for greatest flexibility, with a separate coefficient for its possible value and its interaction with mobility. The coefficient on the government response terms represents its direct impact on transmission, while its interaction represents the degree to which it reduces the strength of the effect of mobility on transmission. The leftmost Column (Columns 1 and 5) in each panel report the share of the sample in which R assumes each of its possible values. The other three columns (Columns 2–4 and 6–8) represent estimates from three regression models with differing controls.

**Table 5:** Regression results relating estimated R values to lagged Google mobility, indicators of strictness of government actions (contact tracing, panel A; facial covering mandates, panel B), and their interactions.

	(1)	(2)	(3)	(4)	(5)	(6)	(7)	(8)
	Panel A: Contact Tracing				Panel B: Facial Covering			
	% Sample	Regression Estimates			% Sample	Regression Estimates		
**Mobility**		0.57***	0.54***	0.42*		0.57***	0.59***	1.00***
		(0.12)	(0.12)	(0.24)		(0.12)	(0.13)	(0.27)
**Action = 1**	33%		-0.10	-0.08	8%		-0.09	-0.20
			(0.06)	(0.10)			(0.07)	(0.12)
**Action = 2**	61%		-0.03	0.02	22%		-0.03	-0.02
			(0.07)	(0.10)			(0.05)	(0.08)
**Action = 3**					46%		-0.11**	-0.24***
							(0.05)	(0.09)
**Action = 4**					21%		-0.07	-0.24**
							(0.06)	(0.10)
**Action = 1 X Mobility**				0.02				-0.54
				(0.24)				(0.42)
**Action = 2 X Mobility**				0.19				0.16
				(0.26)				(0.28)
**Action = 3 X Mobility**								-0.57**
								(0.28)
**Action = 4 X Mobility**								-0.79**
								(0.31)
**Observations**		4782	4642	4642		4782	3978	3978
**Adjusted R-squared**		0.463	0.460	0.460		0.463	0.497	0.512

Government action is encoded in integer values, with 0 (omitted category) indicating no action, and the numbers 1–4 indicating increasingly stricter policy (see text for details). In each panel, the left column (Columns 1 and 5) reports the share of the sample belonging to each value of the strictness indicator. Each of the columns 2–4 and 6–8 report results from a separate regression. All regressions include country fixed effects and week fixed effects. Standard errors, clustered by country, are reported in parentheses. Stars indicate statistical significance (* p<0.1,** p<0.05,*** p<0.01).

**Fig 6:**
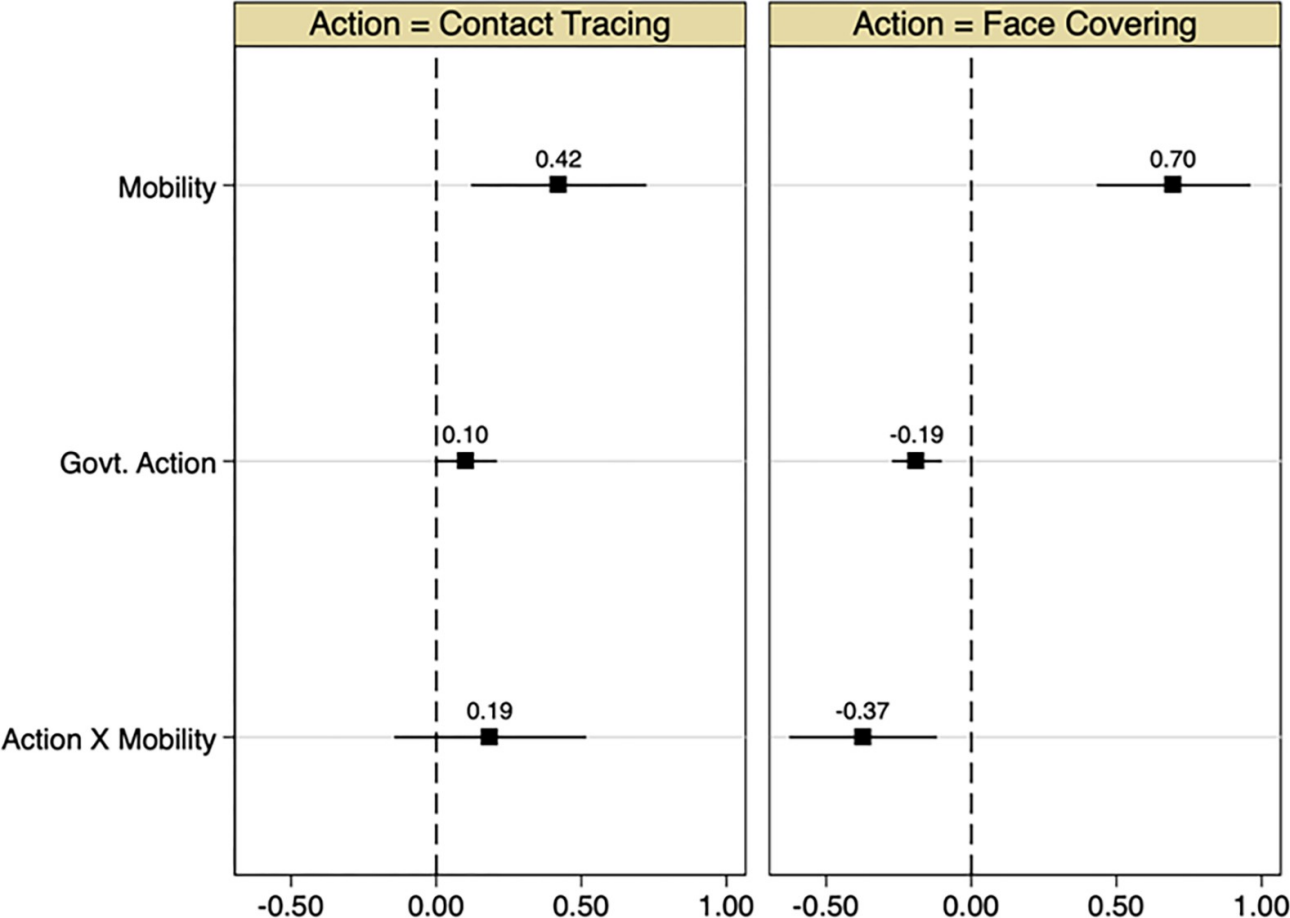
Coefficients from regressions of estimated effective reproduction numbers R(t) observed at the country-week level on Google workplace mobility indicators, indicators of aggressive government action and their interaction. Left panel: Government action is defined as comprehensive contact tracing (in relation to no or limited tracing). Right panel: Government action is defined as requiring face covering is worn in all situations of gathering outside the home (in comparison to weaker or no requirement). See text for details). All regressions include country and week fixed effects. Markers indicate point estimates and error bars indicate 95% confidence intervals corresponding to standard errors that are clustered at the country level.

We also summarize these estimates in Fig 6, where, for simplicity, we reduce each R to a binary variable separating “low” and “high” values. For contact tracing, we choose high values to include the value 2 (comprehensive tracing) and above, and for facial coverings, we choose high values to include the values 3 and 4 (in which coverings are required in all, rather than some shared/public spaces outside the home). The results indicate opposing findings for the two types of responses. Contact tracing does not show a direct significant beneficial effect on transmission, and also does not seem to reduce the effect of mobility. In contrast, sweeping facial covering rules reduce transmission rates by 0.2, and also reduce the strength of the mobility-transmission relation by more than half of its size. In the absence of facial covering rules, a 10 p.p. increase in mobility increases transmission rates by 0.07. When such rules are imposed, the effect of the same mobility increase reduces by 0.037.

## Discussion

Understanding the determinants of Covid-19 transmission rates is one of the most pressing policy questions facing society. This paper provides an empirical analysis of this question, utilizing comprehensive data at global scale and panel data econometric methods to analyze the correlation between mobility levels and transmission rates over time within countries. As such, it provides an important complement to detailed epidemiological modeling of the spread of Covid-19.

Several aspects of our approach are worth noting. As in every cross country study (in contexts not limited to Covid-19 transmission), we analyze relations between country-wide population-weighted averages of variables which may also exhibit intra-country variation. Econometric theory posits that if a certain relation holds between these variables at the local (sub-national) level, it will be recovered by the country-level analysis, albeit with potentially lower statistical precision related to the smaller sample size. If the local relation varies in magnitude sub-nationally, the country-level analysis will recover the average magnitude of the effect.

Additionally, there are significant challenges in interpreting the transmission-mobility relation we estimate. The limited precision of transmission indicators may introduce substantial measurement error into the data, making it hard to identify a correlation. Second, changes in mobility may also endogenously respond to infection rates. Third, variation in mobility levels may be correlated with variation in other forms of preventative behavior, whether voluntary or government dictated, or with environmental factors [22], meaning that any observed correlation can be wrongfully attributed to changes in mobility levels.

We employ standard panel data techniques to address this challenge. In particular, we base our estimates only on variation in mobility and transmission that occur within countries over time, rather than on variation across countries, which is especially prone to omitted variable bias (e.g. economic development levels, infrastructure, health system functionality). Further, we include various forms of flexible temporal trends in our regressions to capture some of the temporal variation in potential confounders that may occur within countries over time. We also note, with the appropriate caution, that many of the potential unobserved confounders would tend to bias our estimates on the relation between mobility and transmission rates upwards, as variation in these confounders likely served to reduce transmission rates in tandem with mobility restrictions (examples include increased mask usage and hygiene).

Our main estimates indicate that overall, a 10 percentage point decline in the Google workplace mobility measure is associated with a 0.05–0.07 unit decline in the estimated reproduction number *R*. Based on these estimates, it is instructive to analyze the share of the overall decline in transmission rates during the early phase of the pandemic (spring) that can be explained by mobility reductions.

Overall, Google mobility rates in Europe declined by approximately 50 percentage points by the beginning of April 2020, which our estimates imply is associated with a reduction in *R* of 0.25–0.35 units. The fraction of the overall decline in *R* that is explained by this mobility reduction depends on the time window chosen for the calculation. By April 11th, the mean value of *R* across the European countries in our sample had declined to 0.95. Given the assumption of a seven-day lag between actual and observed R in the international data, the values corresponding to a given date refer to the R estimated seven days afterwards. Taking the starting date for the calculation as February 21st, when *R* = 3.1, implies an overall decline in *R* of 2.2 units, meaning that mobility reductions explain about 12%-16% of this decline. However, data from February is still very sparse, and derived from only a few countries. If we more conservatively choose the starting time of the calculation to be a month later, on March 11th, when *R* = 2.1—at this point data is available from more than 20 countries, and most of the European mobility reduction has yet to take place—the overall reduction in *R* is reduced to 1.1 units, so that mobility reductions explain approximately 22%–30% of the decline in *R*.

Analogously, in countries within the “Asia and Pacific” region the decline in average Google mobility measures—approximately 40 percentage points to date—imply a reduction of 0.24–0.36 units in *R*. Again, the fraction of the overall decline in *R* explained by mobility depends on the time frame chosen. Taking the starting date of the calculation to be March 11 (average *R* = 1.7), and given the April 11th average value of *R* of approximately 1.27, we obtain that mobility reductions in Asia explain approximately 30%-41% of the reduction in *R*.

These back of the envelope calculations suggest that mobility reductions by themselves played an important role in reducing transmission in the early phases of the pandemic, but they were far from being the only, or even dominant factor. In fact, similar calculations would indicate that in order to reduce the value of R(t) from a typical level of 1.5 during a wave to a stable level of R = 1 solely through mobility reductions, it would be necessary to reduce mobility by approximately 80 percentage points. This is important to note, given the enormous economic costs associated with such reductions. It is also worth keeping in mind that our estimates most likely exaggerate the contribution of mobility restriction, insofar as they are correlated with other forms of behavior change that might reduce transmission.

Our results also speak to the beneficial impact of government interventions such as facial coverings in easing the tradeoff between the economic cost of mobility limits and the health costs of increased transmission rates. For example, given a transmission rate of R = 0.9, our estimates indicate that when facial coverings are in widespread use, mobility can increase by approximately 30 percentage points prior to R exceeding the threshold level of 1. In contrast, when facial masks are not prevalent, mobility can only increase by approximately 15 percentage points prior to exceeding the threshold level.

Changes in the strength of the relation between mobility and transmission over the course of 2020 are also noteworthy. Our estimates suggest the relation was positive and strong in most regions during the spring period, but then declined over the summer, especially in Europe and Asia. Remarkably, however, during the fall and early winter, the magnitude of the relation essentially recovers its original strength in Europe, but remains low in Asia. This disparity may reflect a failure to implement and sustain the lessons of the first spring wave of the pandemic in Europe, vis-a-vis Asia.

Related to that, we also find evidence that facial coverings rules were able to reduce the strength of the relation by more than half of its size, but that contact tracing showed no such effectiveness. This latter failure may be a result of the difficulty of accurately capturing the efficacy of contact tracing implementation across countries in the OxCGRT data. It may well be that this efficacy varies considerably across countries which formally implement such measures comprehensively, at least on paper. It is also known that when the number of new cases is large, such contact tracing programs become very difficult to implement effectively. In contrast, the effectiveness of facial covering rules we estimate is remarkably high, especially given its low economic costs. This effectiveness offers an important lesson for policy makers attempting to strike a balance between trying to contain a pandemic and minimizing its economic impacts.

## Supporting information

S1 File All supporting information is available as a single supplementary file. (DOCX)

S2 File (DTA)

S3 File (DO)
