# Supplementary material for: Correlations of mobility and Covid-19 transmission in global data
Source: PLoS One. 2023 Jul 19;18(7):e0279484. doi: 10.1371/journal.pone.0279484 (PMC10355416; doi:10.1371/journal.pone.0279484)
Supplement: S1 File — (DOCX) [file pone.0279484.s001.docx]

Supporting Information

**Correlations of Mobility and Covid-19 Transmission in Global Data**

Nittai K. Bergman and Ram Fishman


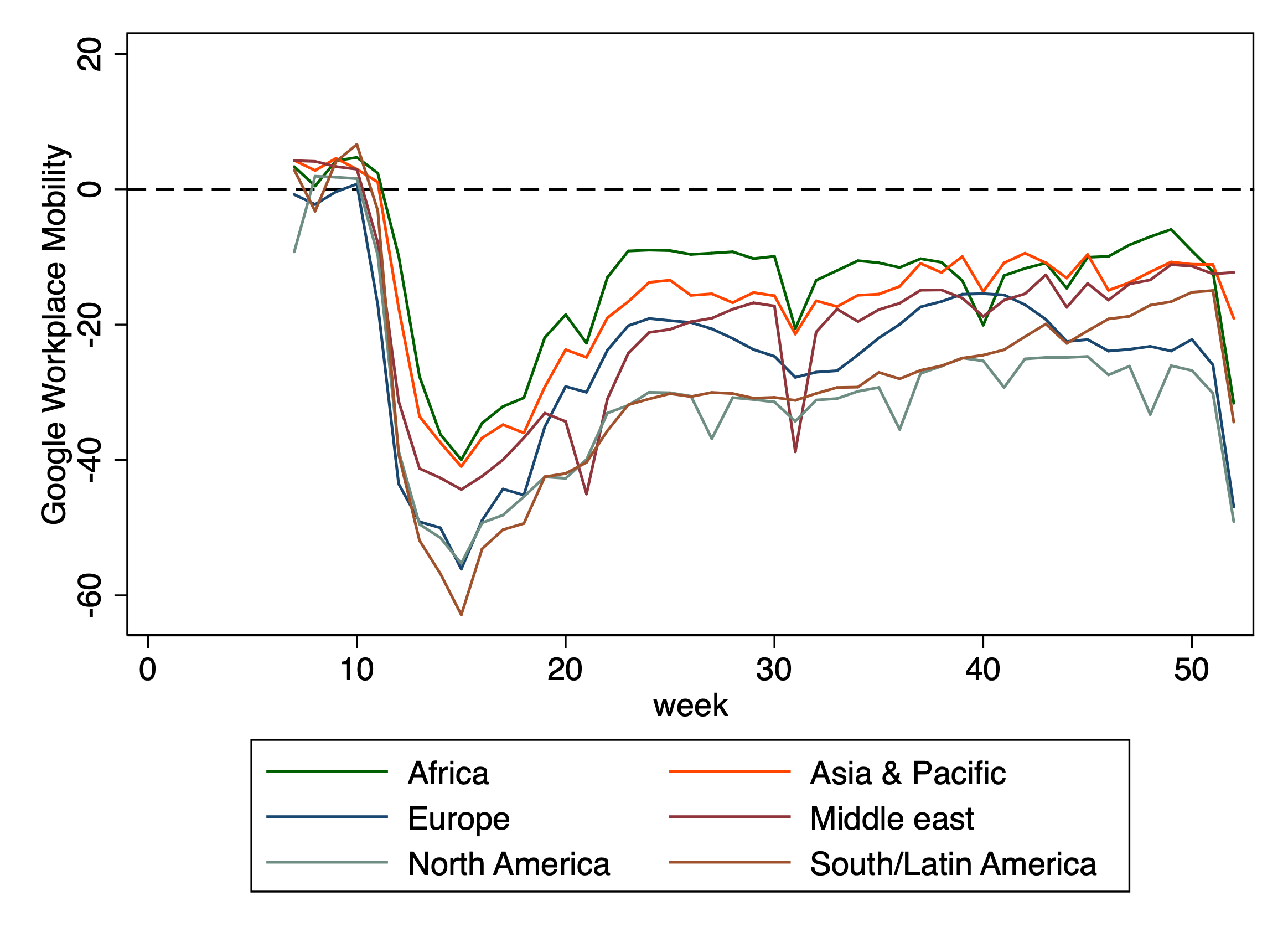


**Figure S1**: Average measure of Google workplace mobility over time, by region. The horizontal axis indicated the week of the year 2021.

**
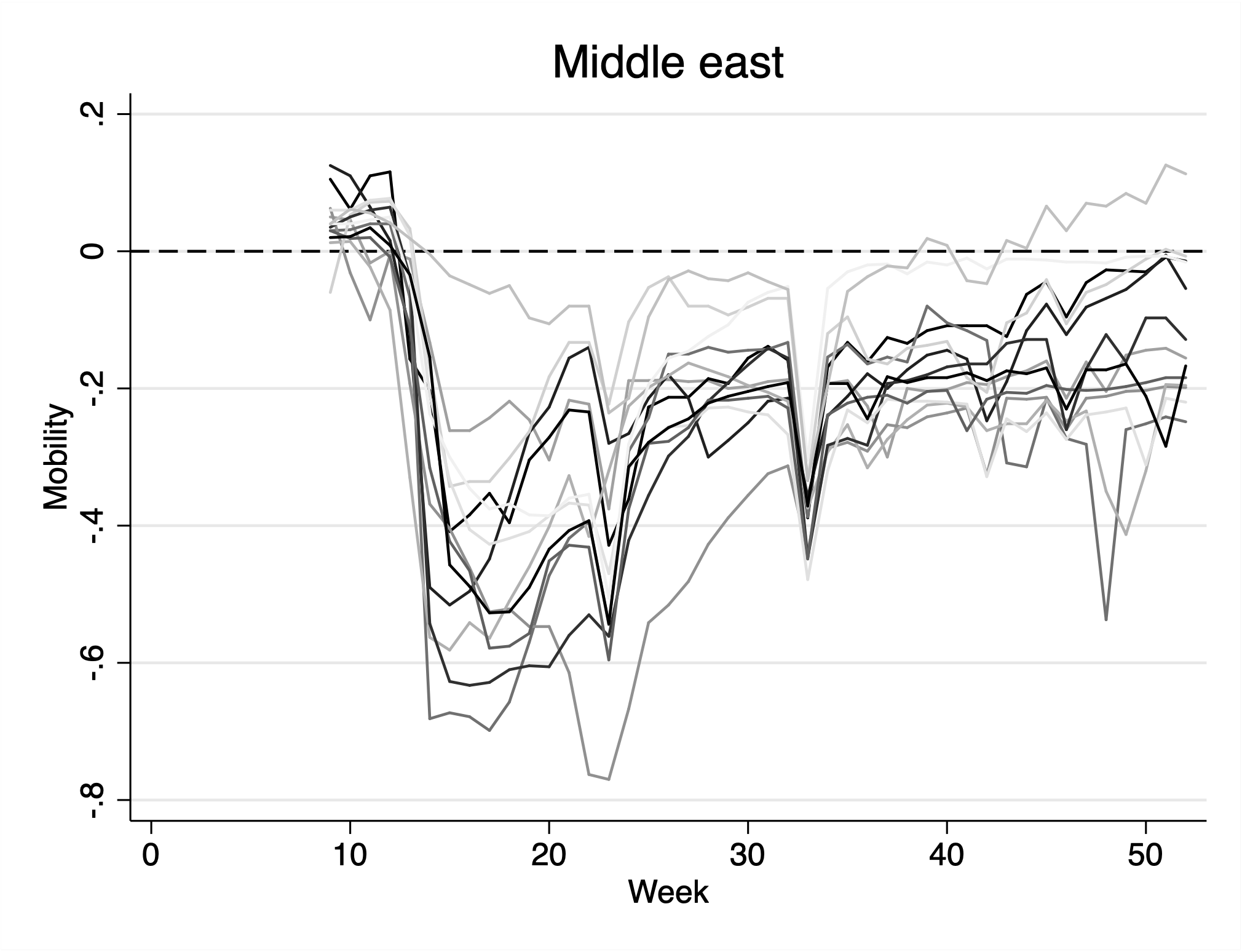

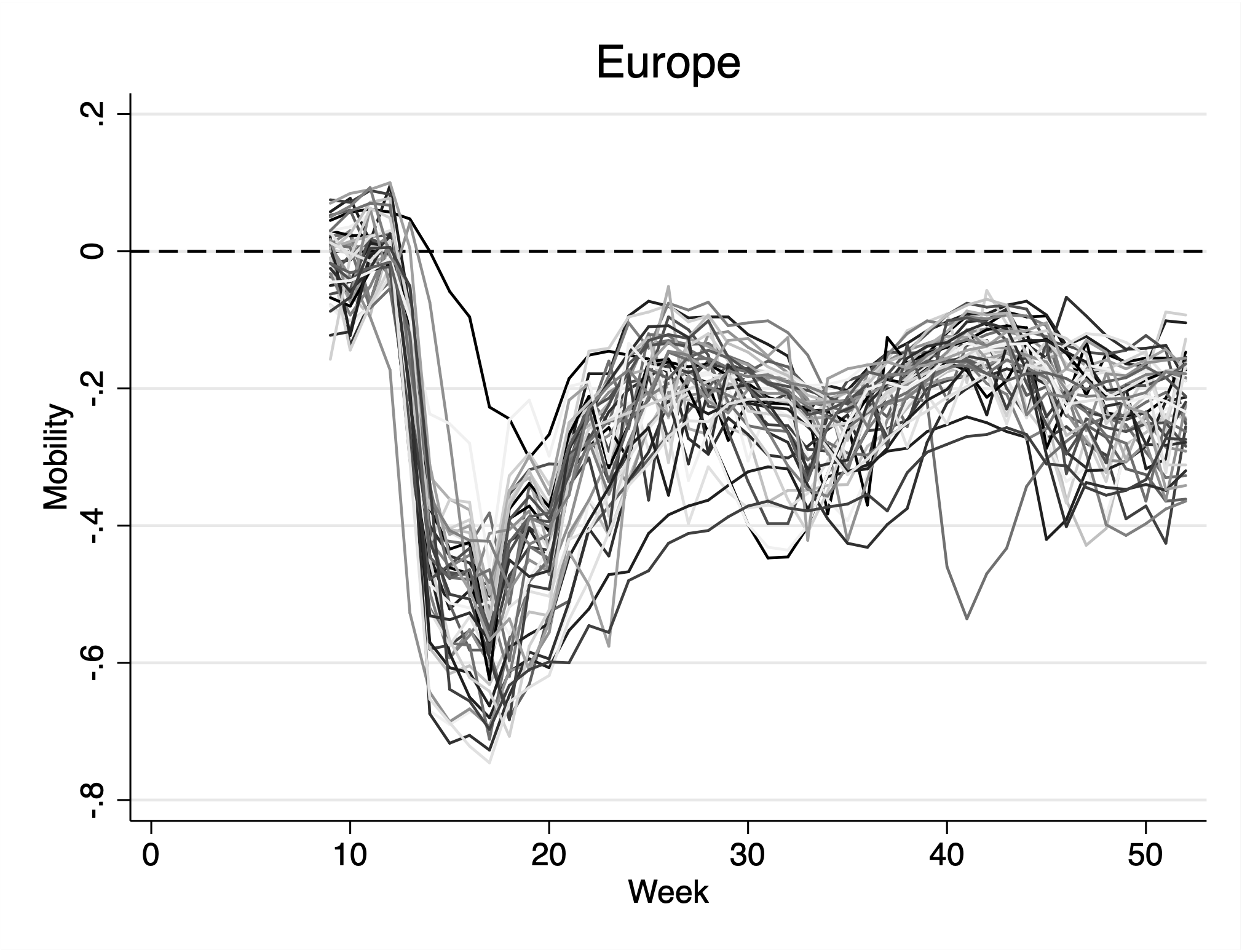

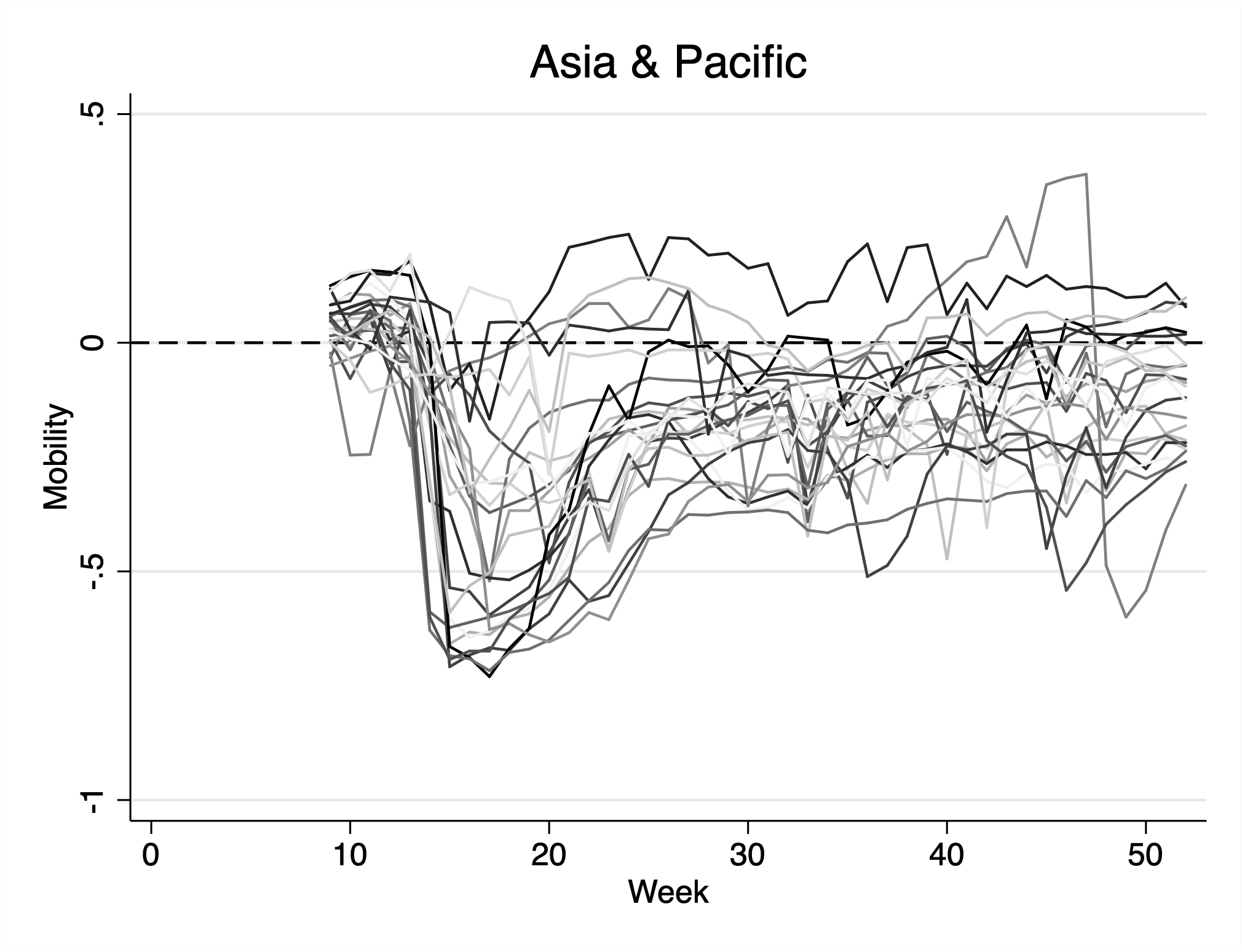

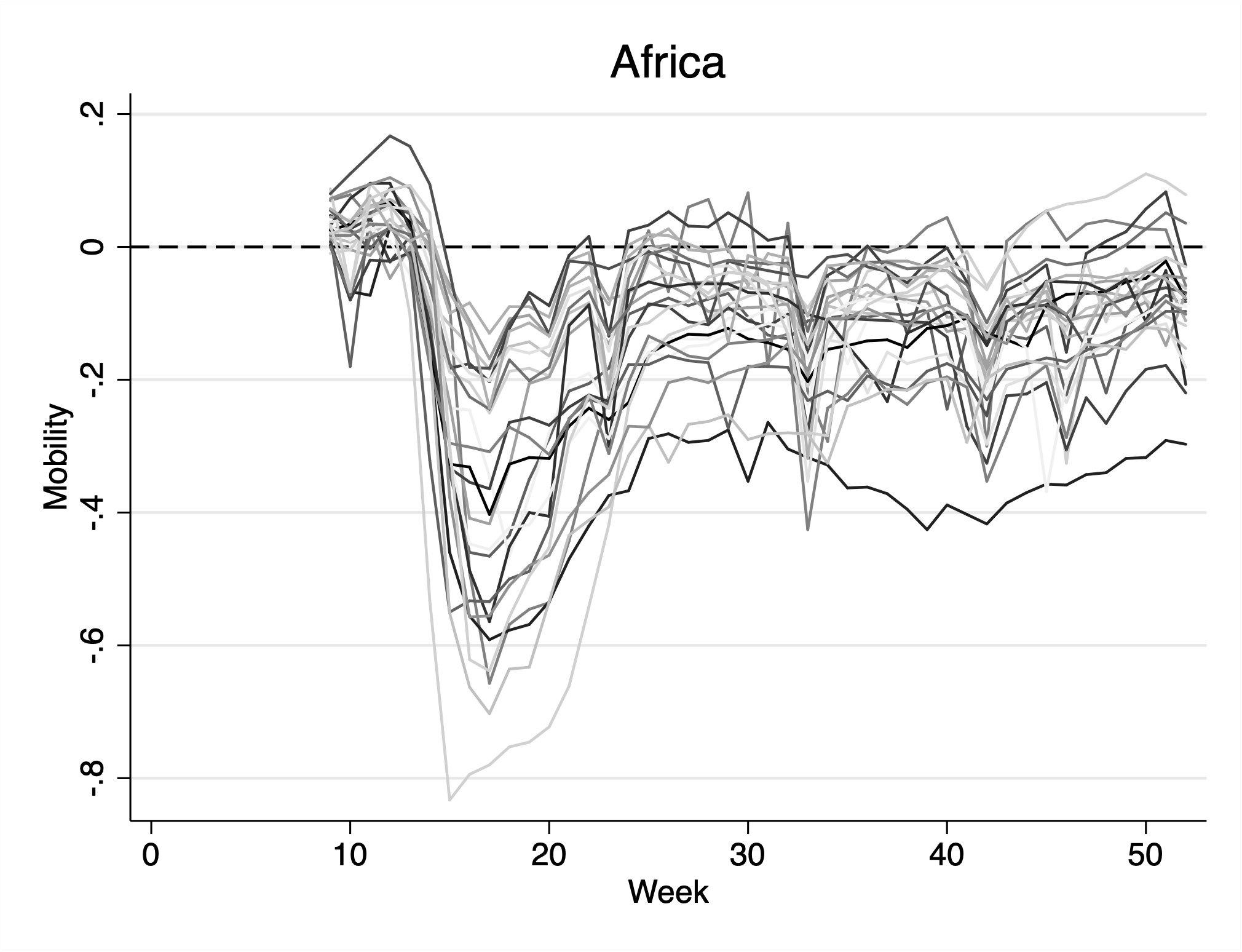
**

**
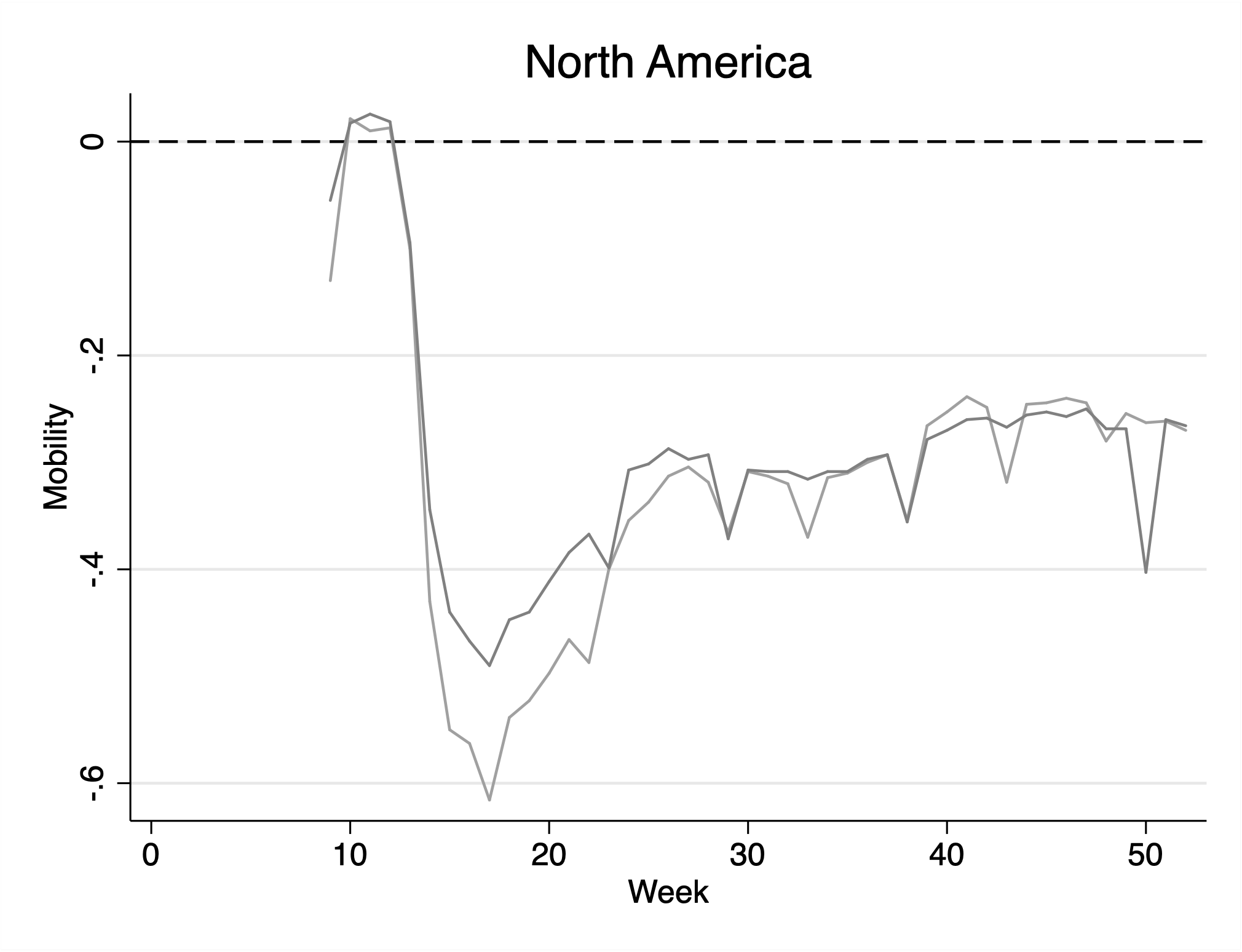
**

**Figure S2**: country level workplace mobility over time, by region. The horizontal axis indicated the week of the year 2021.


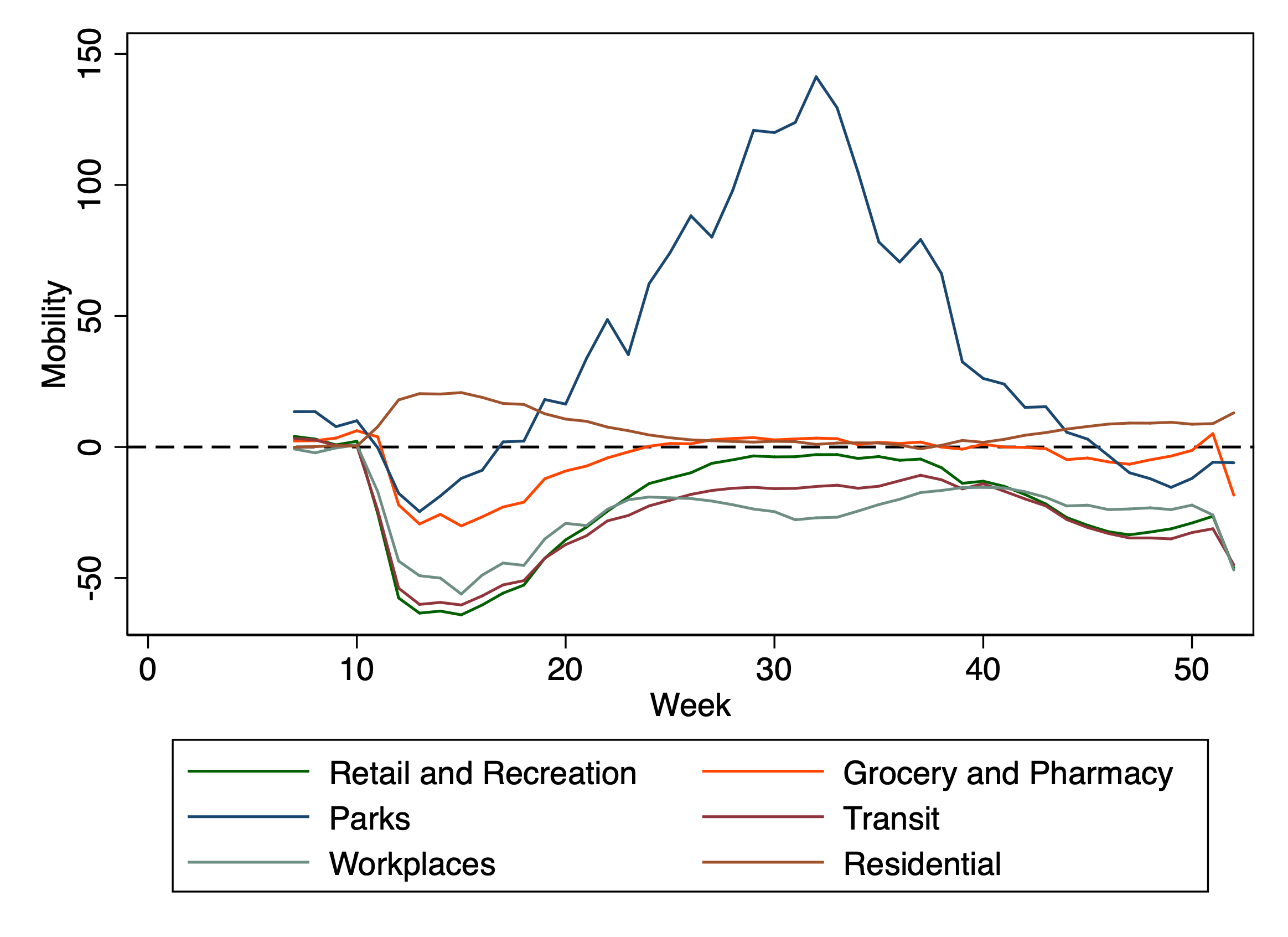


**Figure S3**: Average measures in Europe of Google mobility data, by mobility indicator type, over time.

**
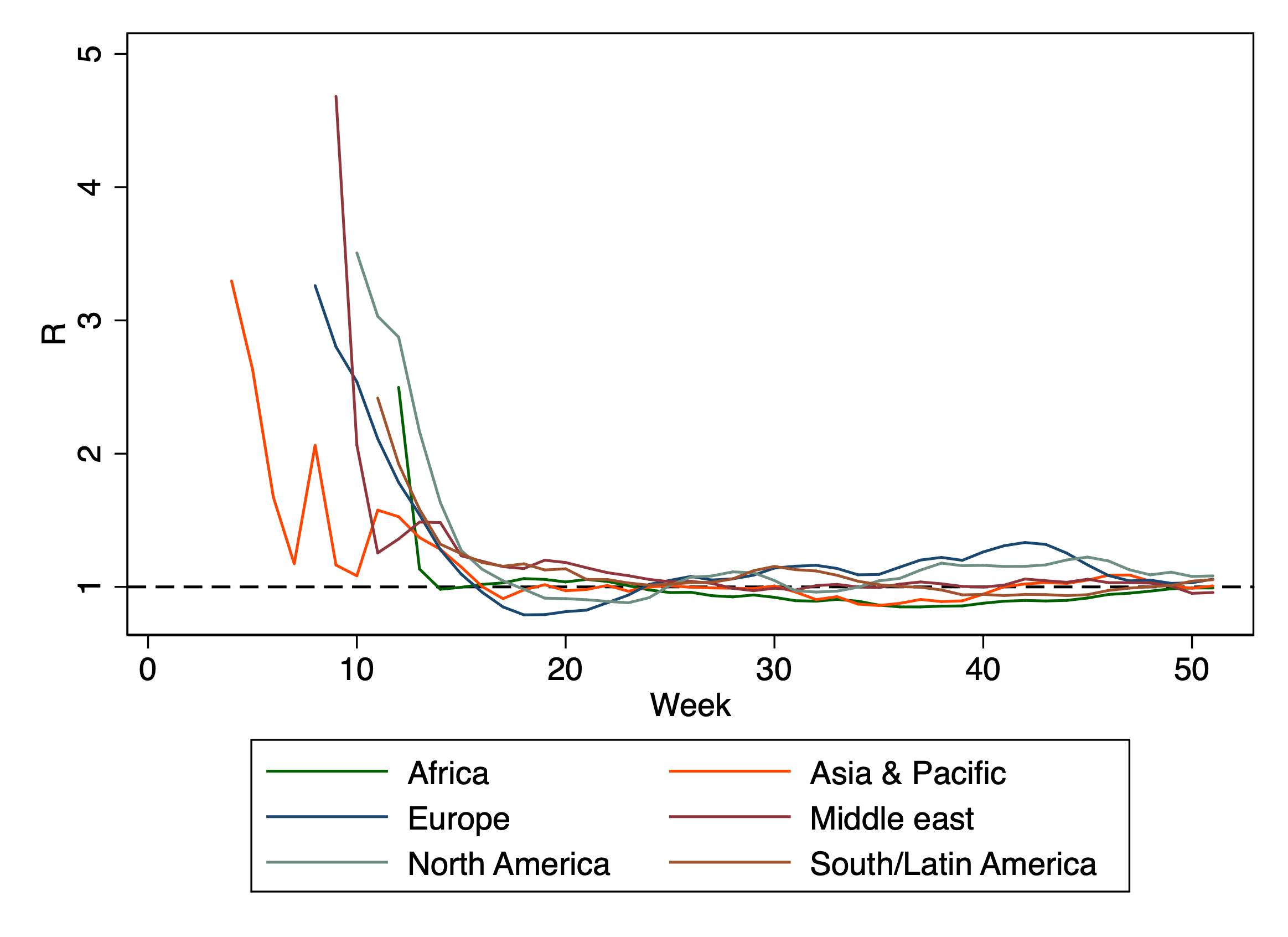
**

**
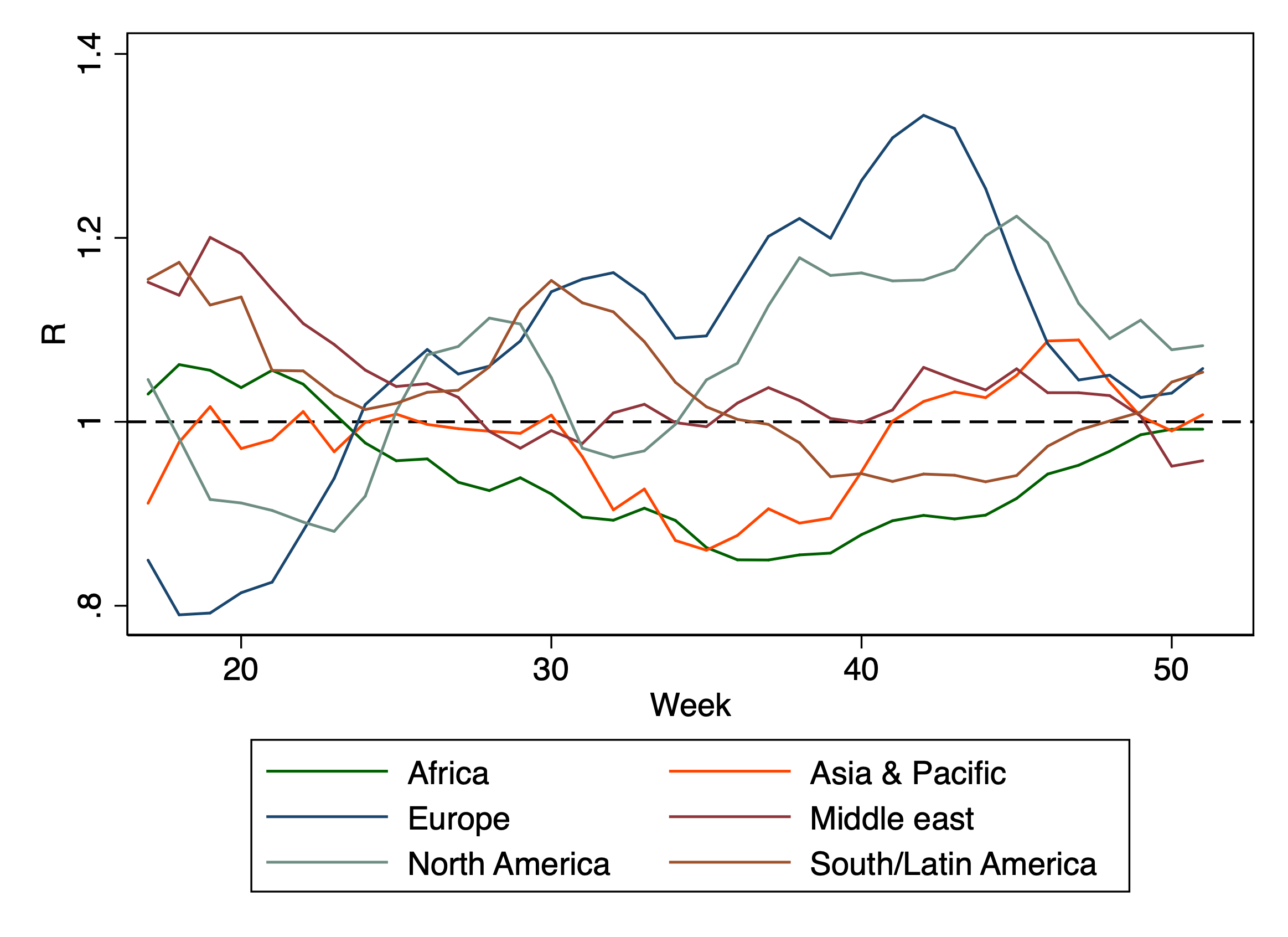
**

**Figure S4:** Regionally averaged estimates of R(t) over time, by region. Top panel: entire period. Bottom panel: The period starting in the first week of April 2020.

**
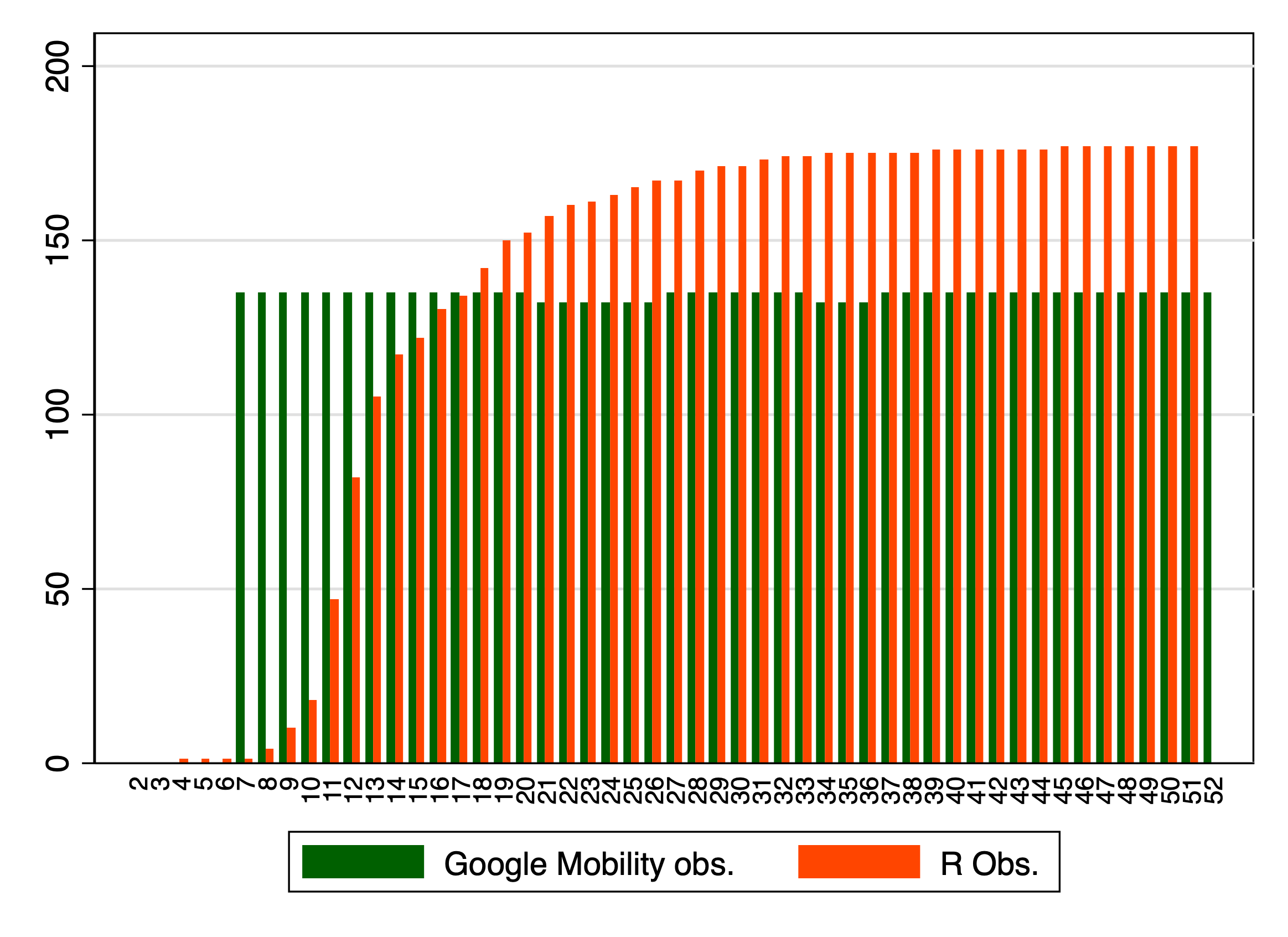
**

**Figure S5:** Number of countries for which mobility and R observations are available, by week.

| **Afghanistan** | **Ecuador** | **Lebanon** | **Poland** | **Uruguay** |
| --- | --- | --- | --- | --- |
| **Angola** | **Egypt** | **Libya** | **Portugal** | **Venezuela** |
| **Antigua and Barbuda** | **El Salvador** | **Liechtenstein** | **Qatar** | **Vietnam** |
| **Argentina** | **Estonia** | **Lithuania** | **Romania** | **Yemen** |
| **Australia** | **Finland** | **Luxembourg** | **Russia** | **Zambia** |
| **Austria** | **France** | **Malaysia** | **Rwanda** | **Zimbabwe** |
| **Bahrain** | **Gabon** | **Mali** | **Saudi Arabia** |  |
| **Bangladesh** | **Georgia** | **Malta** | **Senegal** |  |
| **Barbados** | **Germany** | **Mauritius** | **Serbia** |  |
| **Belarus** | **Ghana** | **Mexico** | **Singapore** |  |
| **Belgium** | **Greece** | **Moldova** | **Slovakia** |  |
| **Belize** | **Guatemala** | **Mongolia** | **Slovenia** |  |
| **Benin** | **Guinea-Bissau** | **Morocco** | **South Africa** |  |
| **Bolivia** | **Haiti** | **Mozambique** | **South Korea** |  |
| **Bosnia and Herzegovina** | **Honduras** | **Namibia** | **Spain** |  |
| **Botswana** | **Hungary** | **Nepal** | **Sri Lanka** |  |
| **Brazil** | **India** | **Netherlands** | **Sweden** |  |
| **Bulgaria** | **Indonesia** | **New Zealand** | **Switzerland** |  |
| **Burkina Faso** | **Iraq** | **Nicaragua** | **Taiwan** |  |
| **Cambodia** | **Ireland** | **Niger** | **Tajikistan** |  |
| **Cameroon** | **Israel** | **Nigeria** | **Tanzania** |  |
| **Canada** | **Italy** | **North Macedonia** | **Thailand** |  |
| **Chile** | **Jamaica** | **Norway** | **Togo** |  |
| **Colombia** | **Japan** | **Oman** | **Trinidad and Tobago** |  |
| **Costa Rica** | **Jordan** | **Pakistan** | **Turkey** |  |
| **Croatia** | **Kazakhstan** | **Panama** | **Uganda** |  |
| **Czechia** | **Kenya** | **Papua New Guinea** | **Ukraine** |  |
| **Côte d'Ivoire** | **Kuwait** | **Paraguay** | **United Arab Emirates** |  |
| **Denmark** | **Kyrgyzstan** | **Peru** | **United Kingdom** |  |
| **Dominican Republic** | **Latvia** | **Philippines** | **United States** |  |

**Figure S6:** A list of the countries included in the main sample


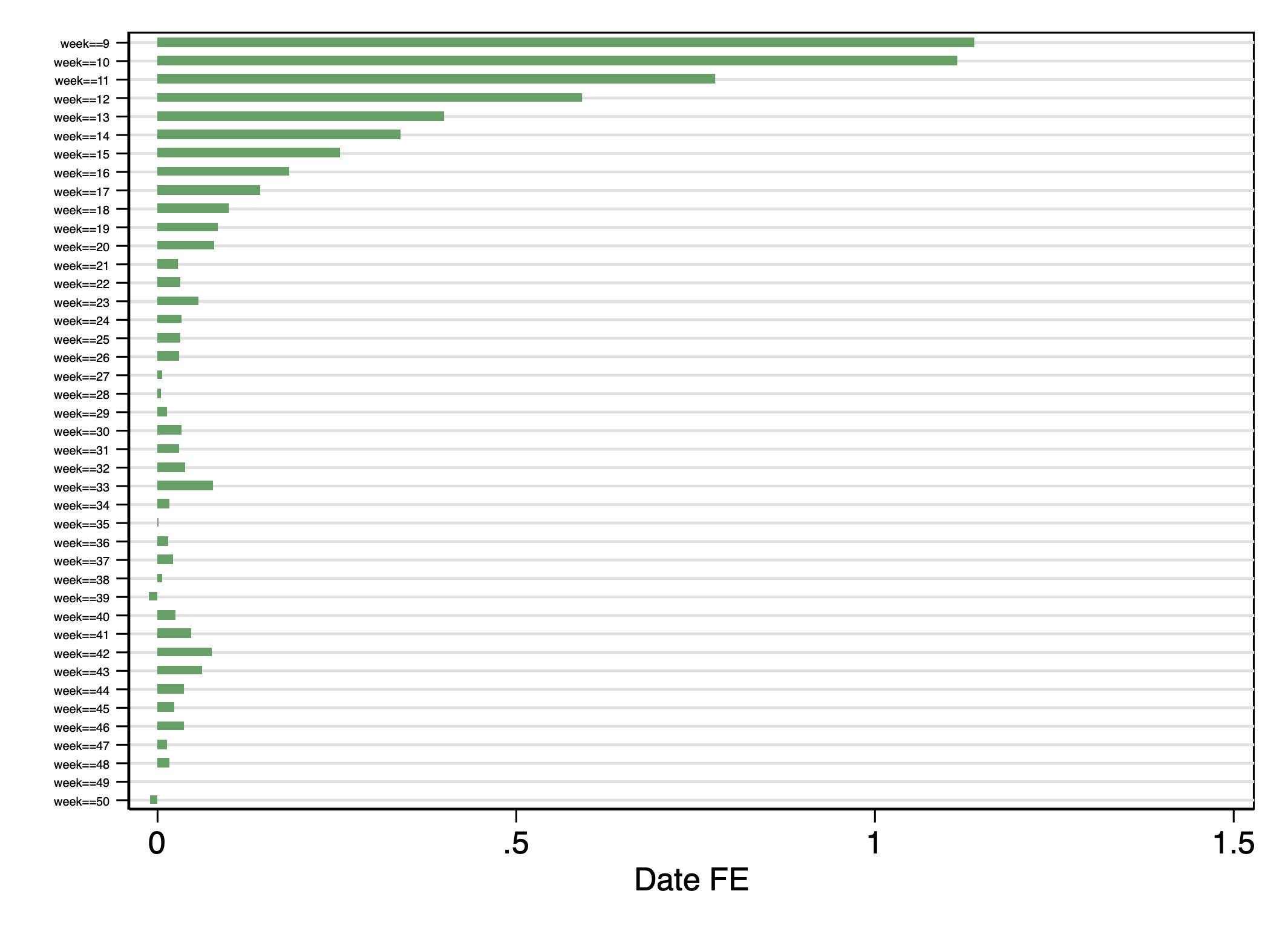


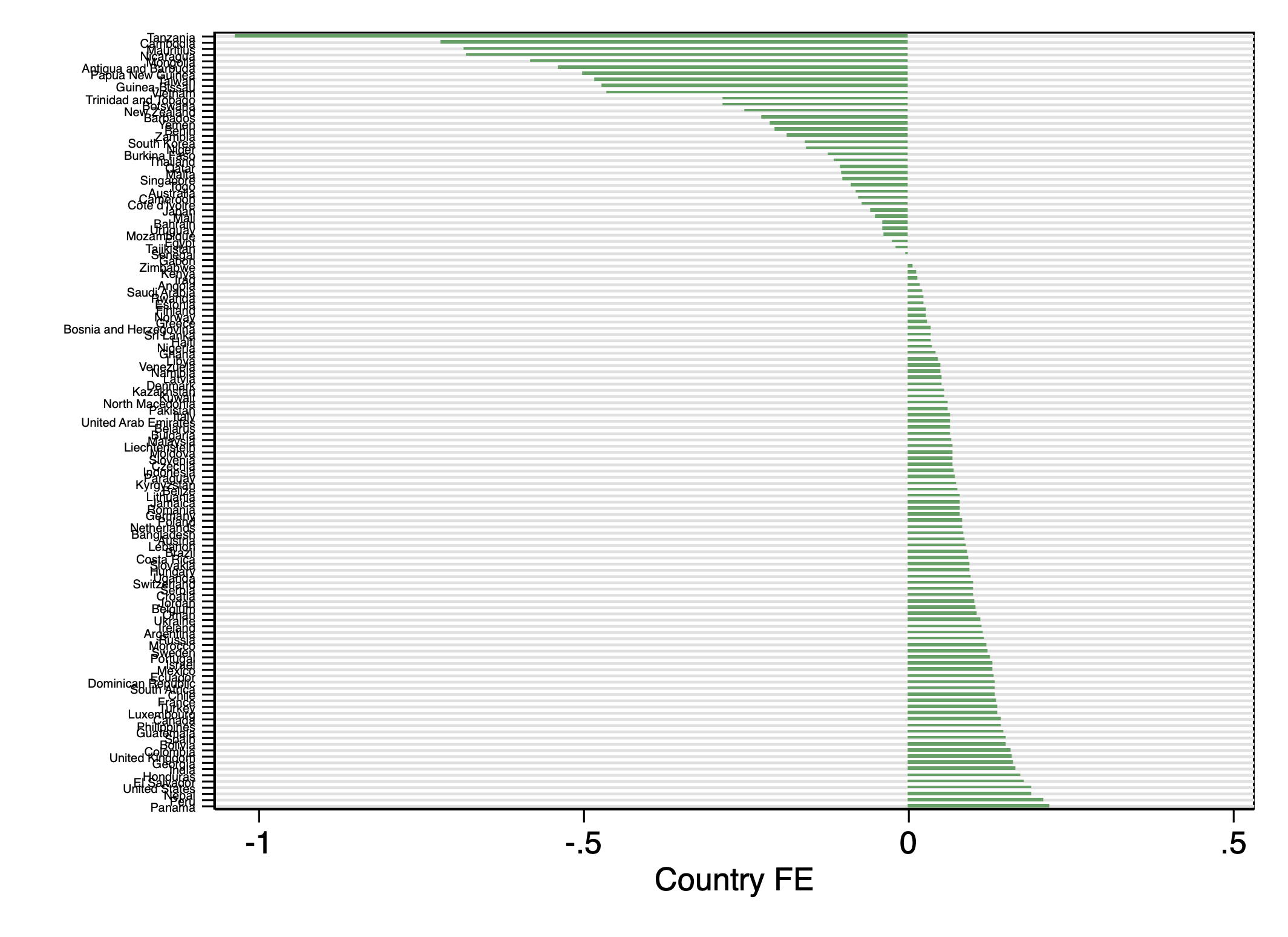


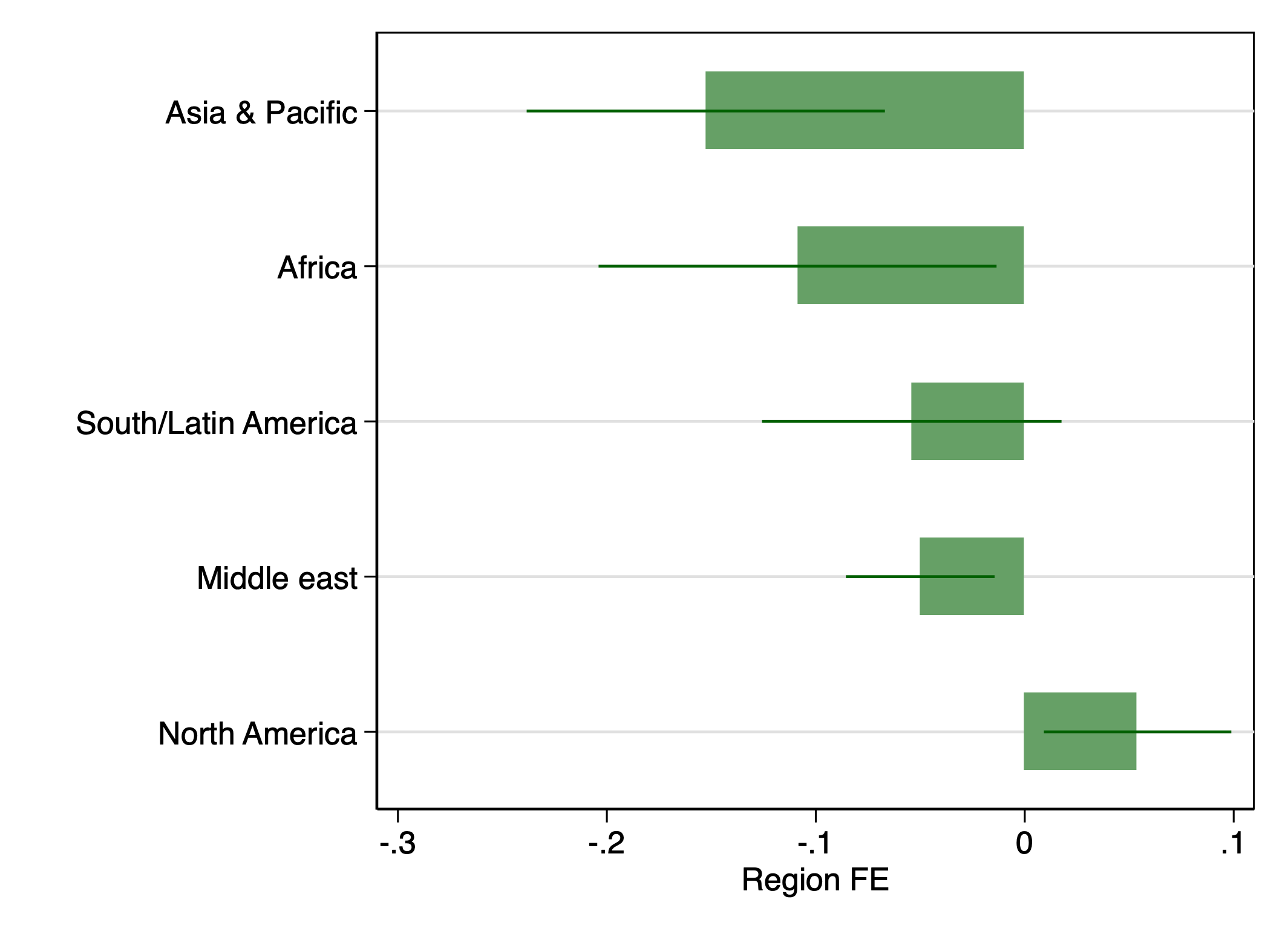


**Figure S7:** Estimated fixed effects for weeks (top, sorted chronologically) and countries (middle, sorted from smallest to largest) from regression (1). Bottom: regional fixed effects from a version of regression (1) which replaces country by region fixed effects, relative to European levels.

**
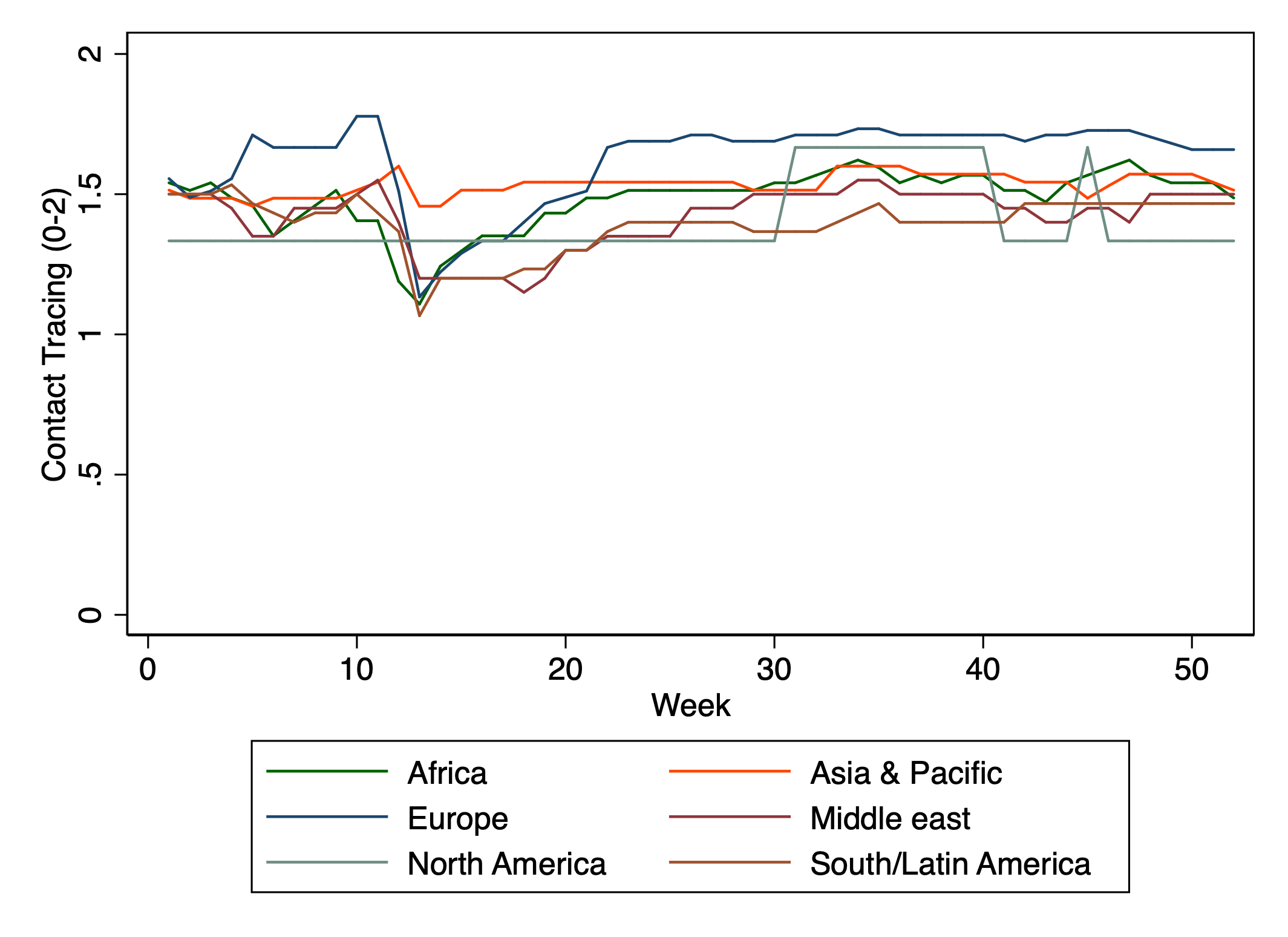

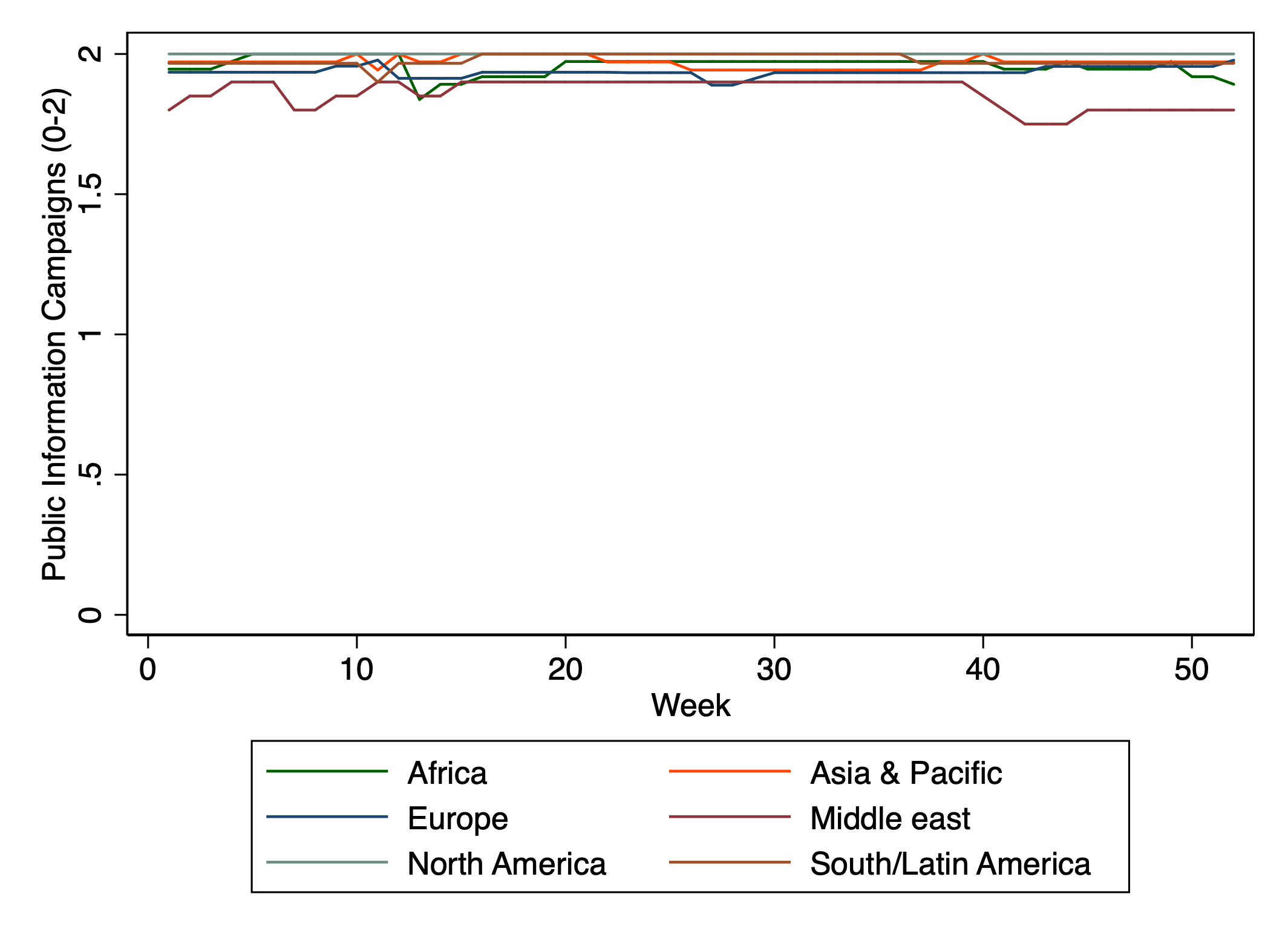

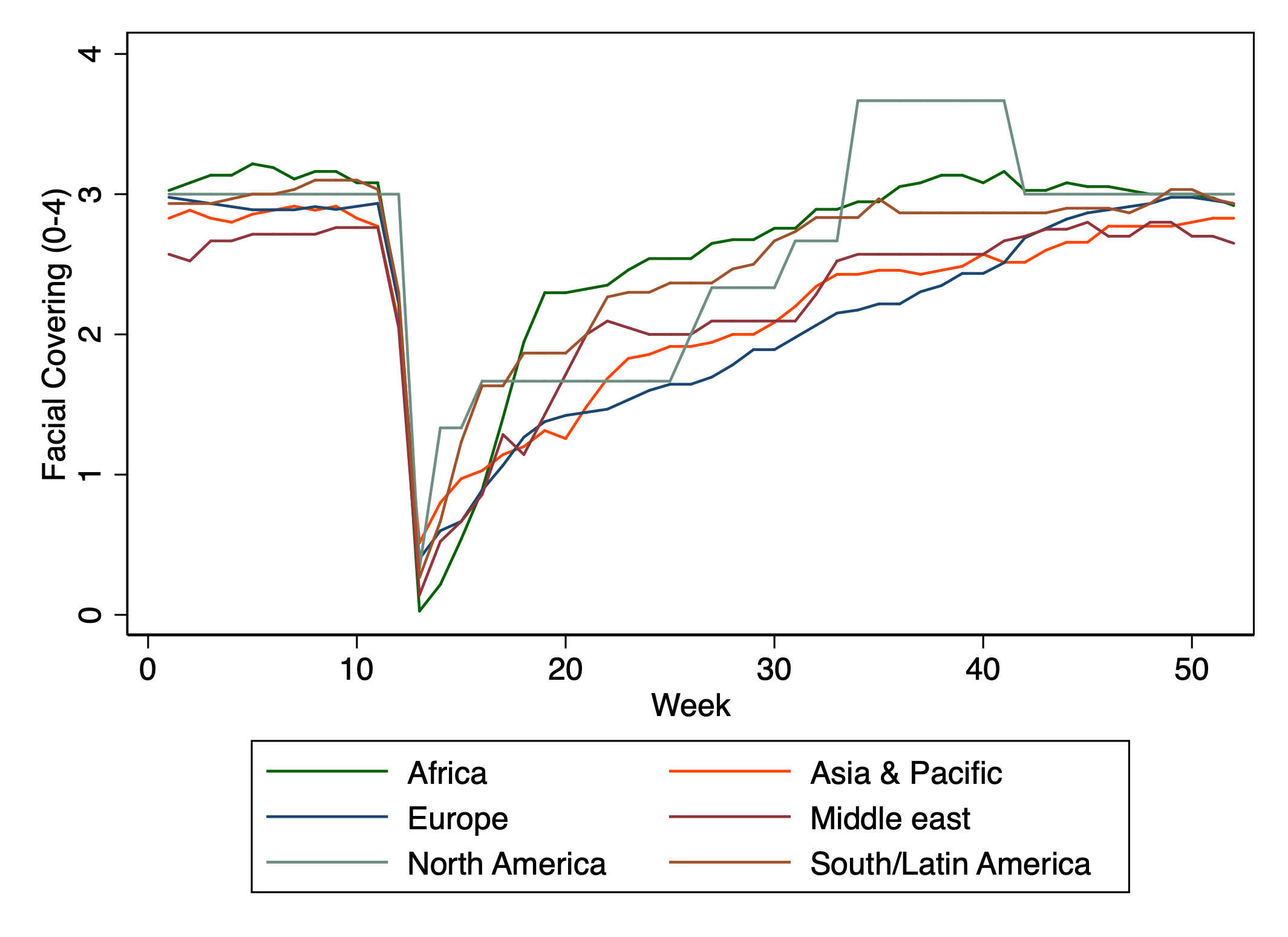
**

**Figure S8**: Average indicators of government actions over time, by region. Top: Facial coverings (0-4). Middle: Contact tracing (0-2). Bottom: Public information campaigns (0-2). Each indicator is measured through an integer number, with 0 indicating absence of action, and higher numbers indicating more aggressive action. Source: Reference 21. For detailed definitions see <https://github.com/OxCGRT/covid-policy-tracker/blob/master/documentation/codebook.md>**.**

**
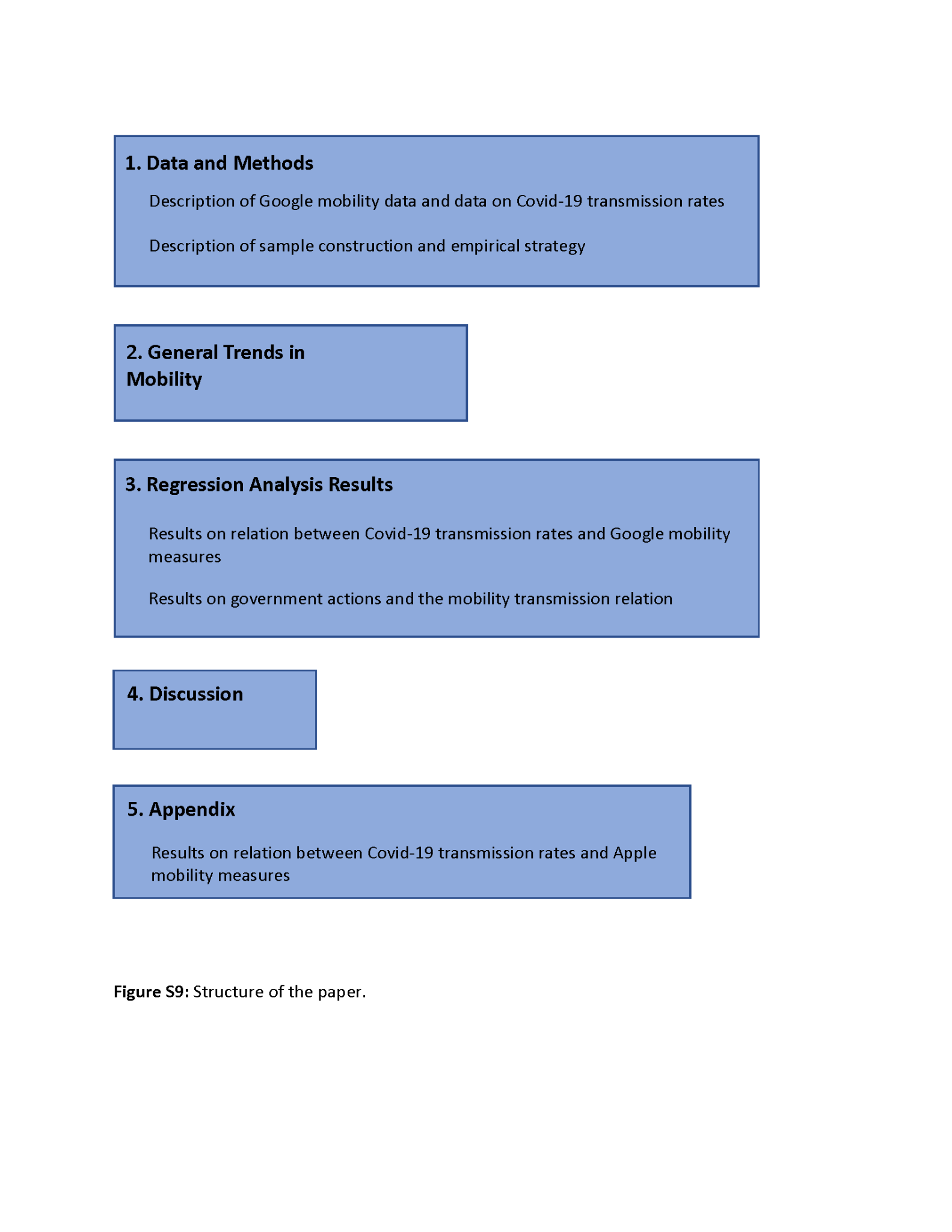
**

|  | (1) | (2) | (3) | (4) |
| --- | --- | --- | --- | --- |
|  | New Cases | Total Cases | New Deaths | Total Deaths |
|  | Growth Rates | | | |
| **L7.Mobility** | 0.13** | 0.11*** | 0.16* | 0.10*** |
|  | (0.05) | (0.02) | (0.10) | (0.04) |
|  |  |  |  |  |
| **Constant** | 1.12*** | 1.13*** | 1.12*** | 1.13*** |
|  | (0.02) | (0.01) | (0.04) | (0.01) |
|  |  |  |  |  |
| **Observations** | 3924 | 4704 | 1165 | 3112 |
| **Adjusted R-squared** | 0.095 | 0.492 | 0.174 | 0.448 |

**Table S1:**  Regression results relating alternative measures of Covid-19 transmission to the 7-day lagged Google workplace mobility indicator. Column 1: dependent variable is the daily growth rate of new cases (3-day running mean). Column 2: dependent variable is the daily growth rate of total confirmed cases. Column 3: dependent variable is the daily growth rate of new deaths (3-day running mean). Column 4: dependent variable is the daily growth rate of total deaths. All regressions include country and date fixed effects. Standard errors, clustered by country, are reported in parentheses. Stars indicate statistical significance (* p<0.1,** p<0.05,*** p<0.01).

**Mobility Data from Apple**. We also use the *Mobility Trends Reports* provided by Apple as a robustness test. These data report the “relative volume of directions requests” sent using the Apple Maps application, compared to a baseline volume on January 13th, 2020. The Apple data differentiate between three types of direction requests: driving, walking, and transit, an is available for 63 countries. The sample used for the Apple data analysis includes 2,503 observations from 61 countries over the period March 13th - Dec 31st.

Table S2 repeats the analysis of Table 1 but uses the Apple driving mobility measure to proxy for mobility levels. Results show a similar pattern, even though the estimates are substantially smaller, reflecting the different definitions of mobility and potentially, the much smaller sample of countries covered by this data.

|  | (1) | (2) | (3) | (4) |
| --- | --- | --- | --- | --- |
|  | **R** | **R** | **R** | **R** |
|  |  |  |  |  |
| Apple Driving Mobility (Seven-day Lagged) | 0.15*** | 0.14*** | 0.13** | 0.17*** |
|  | (0.04) | (0.05) | (0.06) | (0.06) |
|  |  |  |  |  |
| Observations | 2503 | 2503 | 2461 | 2461 |
| Adjusted R-squared | 0.124 | 0.485 | 0.544 | 0.545 |
|  |  |  |  |  |
| Week F.E. | None | Global | Regional | Global |
| Weeks Since 100th Case F.E. | N | N | N | Regional |

**Table S2**: Regression results relating estimated *R* values to 2 week lagged Apple driving mobility. Each column reports results from a separate regression. All regressions include country fixed effects. Standard errors, clustered by country, are reported in parentheses. Stars indicate statistical significance (* p<0.1,** p<0.05,*** p<0.01).
